# GCN2 inhibition reduces mutant SOD1 clustering and toxicity and delays disease progression in an amyotrophic lateral sclerosis mouse model

**DOI:** 10.1186/s40035-024-00441-w

**Published:** 2024-09-20

**Authors:** Didio Alberto Ortiz, Nuria Peregrín, Miguel Valencia, Rodrigo Vinueza-Gavilanes, Elisa Marín-Ordovas, Roberto Ferrero, María Jesús Nicolás, Gloria González-Aseguinolaza, Montserrat Arrasate, Tomás Aragón

**Affiliations:** 1 DNA and RNA Medicine Program, Center for Applied Medical Research (CIMA), University of Navarra, Pamplona, Spain; 2 Biomedical Engineering Program, Center for Applied Medical Research (CIMA), University of Navarra, Pamplona, Spain; 3 Institute of Data Science and Artificial Intelligence (DATAI), University of Navarra, Pamplona, Spain; 4 Navarra Institute for Health Research (IdiSNA), Pamplona, Spain; 5 Neuroimmunology Department, Multiple Sclerosis Center of Catalonia (Cemcat), Vall d’Hebron Research Institute (VHIR), Barcelona, Spain; 6 School of Medicine, University of Navarra, Pamplona, Spain; 7 Present Address: Brain Lab, Biobizkaia Health Research Institute, Barakaldo, Spain; 8 Present Address: Ikerbasque, Basque Foundation for Science, Bilbao, Spain

## Main text

The disruption of protein folding homeostasis in motoneurons (MNs) and the accumulation of protein aggregates are some of the main molecular hallmarks of amyotrophic lateral sclerosis (ALS). Evidence from sporadic and familial ALS (fALS) patients and from ALS models suggests that protein aggregation directly participates in neurodegeneration. In turn, the loss of MN homeostasis triggers a coping mechanism, the integrated stress response (ISR) [1]. The ISR is initiated by four independent stress-sensing kinases, each of them activated by distinct stresses: protein kinase R (PKR) by double-strand RNA, protein kinase RNA-like endoplasmic reticulum kinase (PERK) by protein misfolding at the endoplasmic reticulum (ER), general control nonderepressible 2 (GCN2) by nutrient starvation, and heme-regulated inhibitor (HRI) by heme deprivation, mitochondrial stress and proteasome deficiency. Once activated, they phosphorylate the alpha subunit of eukaryotic initiation factor 2 (eIF2a), leading to (1) a general reduction in translation of most mRNAs and (2) an enhanced translation of transcripts encoding stress response factors, like the ATF4 transcription factor. While these gene expression changes are in principle neuroprotective, under chronic stress the ISR can drive apoptosis [1]. In most ALS studies, the ISR kinase PERK was proposed as the main ISR trigger. Importantly, in ALS animal models ISR activation is most prominent in fast-fatigable MNs, the most vulnerable MN subpopulation [2]. While pharmacological ISR activators or suppressors have been developed to treat ALS [3], it is still unclear what stress(es) drive ISR activation in ALS and which is the most robust therapeutic strategy.

Mutations in the *SOD1* gene cause fALS and SOD1 protein aggregation. We previously developed a neuronal ALS model based on the expression of the mutant allele *SOD1* G93A, that recapitulates ISR activation and where the risk of neuronal death can be quantitatively scored. In this model, the ISR downstream inhibitor (ISRIB) [4] tuned neuronal ISR and enhanced neuronal survival [5]. Intriguingly, ISRIB also mitigated a distinct proteostatic mechanism, the unfolded protein response [5]. The neuroprotective effect of ISR inhibition was not recapitulated by PERK inhibition [5], suggesting that ISR stress-sensing kinase(s) other than PERK trigger ISR activation in ALS.

**Fig. 1:**
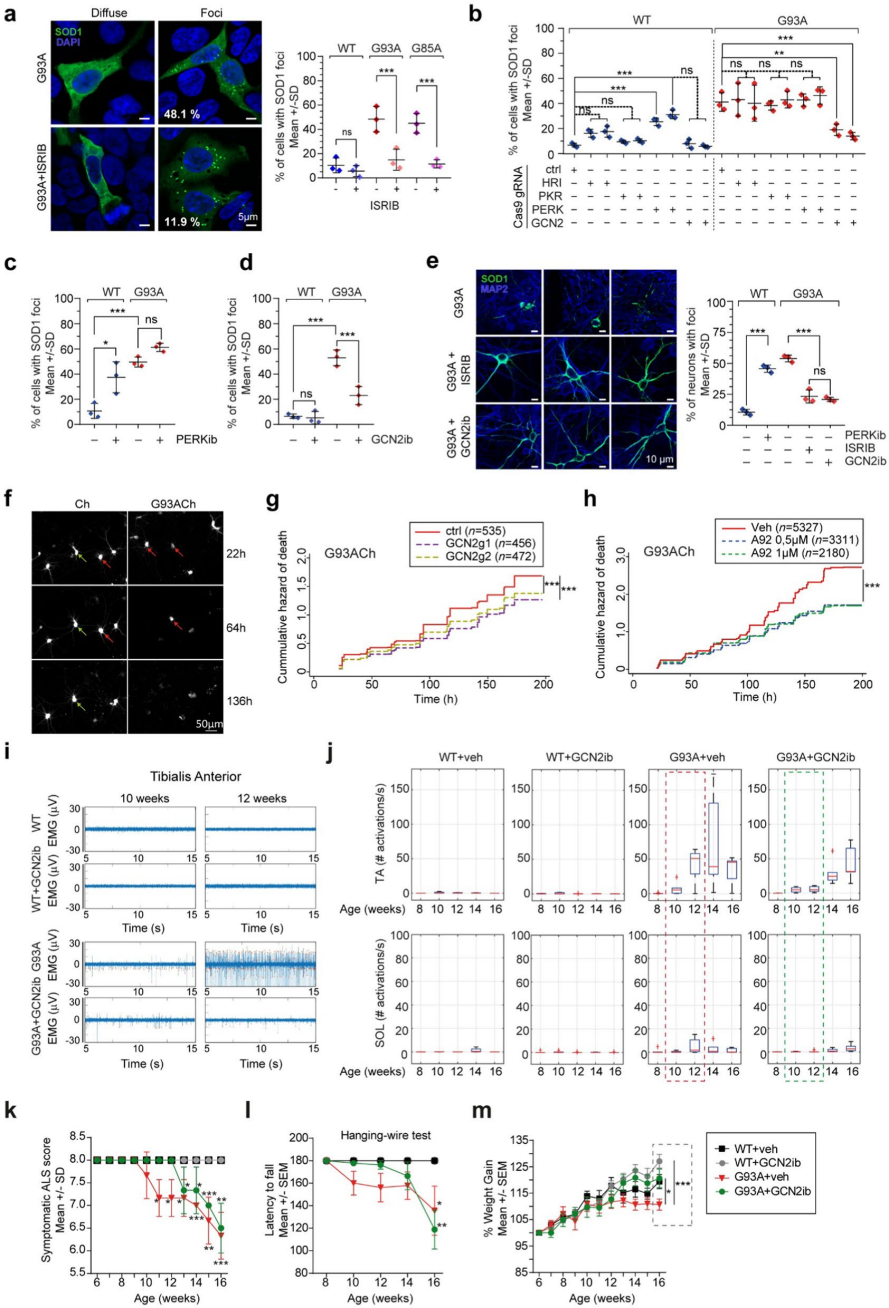
**a** SOD1 immunofluorescence in HEK293 cells overexpressing mutant SOD1; percentage of cells with SOD1 foci. **b** HRI, PKR, PERK or GCN2 knock-down effect on the percentage of HEK293 cells with SOD1 foci. HEK293 cells co-transfected with plasmids expressing SOD1 (WT or G93A) and the Cas9 endonuclease with gRNAs targeting *HRI*, *PKR*, *PERK* or *GCN2* or a non-targeting gRNA (control). **c**,** d** PERK and GCN2 pharmacological inhibition (PERKib, GCN2ib) effect on SOD1 (WT, G93A) foci formation in HEK293 cells. FociCount SOD1 foci analysis (Fig. S2), 3 independent experiments, ≥ 100 cells/condition/experiment, one-way ANOVA and Sidak´s *post-hoc* test. **e** SOD1 and MAP2 immunofluorescence in primary neurons expressing mutant SOD1 +/-ISRIB or GCN2ib ; clustered distribution pattern quantification. One-way ANOVA and Sidak´s post hoc test, 3 independent experiments, > 25 neurons/condition/experiment. **f** Longitudinal tracking of neurons expressing mCherry (Ch) and Ch-tagged mutant SOD1 (G93ACh). Green arrows point to neurons tracked till the end of the experiment. Red arrows indicate neurons that died before. **g** Cumulative death hazard of neurons co-expressing G93ACh or Ch and gRNAs-containing CRISPR/Cas9 plasmids for GCN2 targeting (GCN2g1, GCN2g2) or px458 empty vector (ctrl). **h** Cumulative death hazard of neurons expressing G93ACh +/- GCN2 pharmacological inhibitor (A92). Cox Proportional Hazard analysis; pooled data from 3–5 experiments (Tables S1-S2). *n*; number of neurons. **i** EMG activations in TA from WT and G93A mice +/- GCN2ib (10 and 12 weeks of age). **j** Longitudinal EMG analysis in TA and SOL from WT and G93A mice +/- GCN2ib. Red/green boxes highlight differences in GCN2ib-treated G93A mice. Two-way Repeated Measures (RM) ANOVA. Statistic analysis details in Additional file 1. Supplementary Statistical Analysis. Median and 25th -75th percentile (“+”indicates outlier). **k–m** Effect of GCN2 inhibition on clinical score, motor phenotype, and weight gain. WT *n* = 4 mice, WT + GCN2ib *n* = 6 mice, G93A *n* = 6 mice, G93A + GCN2ib *n* = 6 mice. Two-way RM ANOVA and Tukey´s *post-hoc* test. Statistic analysis details in Additional file 1. Supplementary Statistical Analysis. *P < 0.05, **P < 0.01, **P < 0.001

To investigate if ISR modulation affects the localization and neurotoxicity of mutant SOD1 protein, we explored the effect of ISRIB on SOD1 clustering. Overexpression of mutant SOD1 in HEK293 cells led to protein cluster formation in approximately 50% of cells (Fig. S1). Using a semi-automated image analysis tool (FociCount) (Fig. S2), we quantitatively confirmed that ISRIB prevented mutant SOD1 clustering (Fig. 1a, Fig. S3a) without changing SOD1 steady-state levels (Fig. S3b).

By relieving the translational inhibition imposed by eIF2a phosphorylation, ISRIB prevents the formation of stress granules (SGs), messenger ribonucleoprotein assemblies formed upon abrupt translational shutdown [4]. Since SGs contribute to the aggregation of FUS and TDP43 ALS neurotoxic proteins [6], we asked whether SOD1 foci were related to SGs. Mutant SOD1 failed to promote the clustering of the SG marker G3BP2 in HEK293 cells (Fig. S3c). Moreover, SOD1 clusters did not colocalize with arsenite-induced SG foci, confirming that SOD1 foci are not SG-related (Fig. S3c). Thus, basal or the stress-induced ISR is necessary for SOD1 clustering.

To identify the ISR kinase(s) required for SOD1 clustering, we generated CRISPR-Cas9 gene editing constructs that prevent the expression of human ISR kinases (Fig. S4c, d) and analyzed their effect on WT and mutant SOD1 clustering. Surprisingly, WT and mutant SOD1 clustering ability was affected by distinct ISR kinases. In the case of WT SOD1, *PERK* gene editing increased the fraction of SOD1-expressing cells containing foci (Fig. 1b and Fig. S5). Differently, *GCN2* gene editing strongly reduced mutant SOD1 clustering ability (Fig. 1b and Fig. S6). These effects were indeed due to the loss of their kinase activity since two GCN2 or PERK kinase inhibitors: PERKib (GSK2606414) and GCN2ib (GCN2iB) had the same effect. As anticipated, PERKib prevented PERK autophosphorylation/activation and ATF4 translation in cells treated with the ER stress pharmacological inducer thapsigargin (Thap) (Fig. S4b). Similarly, upon treatment with the histidine analog histidinol (HisOH), GCN2ib prevented GCN2 autophosphorylation and the ensuing translation of ATF4 protein. These effects were specific since neither of the inhibitors prevented the activation of the non-cognate ISR kinase (Fig. S4b). In line with the CRISPR/Cas9-based observations, PERKib enhanced the appearance of WT SOD1 foci and tended to increase the number of cells with SOD1G93A foci (Fig. 1c, Fig. S7a), while GCN2ib strongly reduced SOD1 clustering (Fig. 1d, Fig. S7b).

Next, we tested if PERK and GCN2 inhibition affected SOD1 distribution in primary neurons. In neurons overexpressing WT SOD1, the protein was evenly distributed through soma and processes; quite differently, mutant SOD1 protein was discontinuously distributed through soma, dendrites, and axons. Treatment of WT SOD1-expressing neurons with PERKib subverted the protein distribution into a discontinuous pattern (Fig. S8), while blocking the ISR with ISRIB or GCN2ib restored the homogeneous distribution of mutant SOD1 (Fig. 1e). These results put forward an unanticipated role of GCN2 in mutant SOD1 behaviour.

The role of GCN2 in mutant SOD1-induced neurodegeneration was analyzed by longitudinal survival analysis (Fig. 1f) [5]. We generated and validated constructs expressing the endonuclease Cas9 with two different guide RNAs (gRNAs) targeting the coding sequence of rat GCN2 (Fig. S4e, f). Then, we co-transfected primary neuronal cultures with these constructs (or a control plasmid expressing a non-targeting gRNA) and plasmids expressing a recombinant version of SOD1G93A bearing a C-terminal mCherry tag (G93ACh) [5]. As an additional control, CRISPR-Cas9 constructs were co-transfected with a plasmid expressing mCherry (Ch). As shown in Fig. 1g, GCN2 knock-down with two different gRNAs enhanced the survival of neurons overexpressing G93ACh, while it did not affect the survival of Ch-expressing neurons (Fig. S9a). Accordingly, pharmacological inhibition of GCN2 (Fig. S9b) reduced the risk of death of G93ACh-expressing neurons (Fig. 1h) and, to a lower extent, the risk of death of Ch-expressing neurons (Fig. S9c). Therefore, GCN2 plays an important role in mutant SOD1 neuronal distribution and toxicity.

Finally, we evaluated the therapeutic potential of GCN2 inhibition by treating the SOD1^G93A^ transgenic ALS mouse model with a pharmacological GCN2 inhibitor. The small molecule GCN2ib was intraperitoneally delivered to WT and G93A male mice at 10 mg/kg (twice a day) from 6 to 16 weeks of age. We tracked the denervation process by detecting spontaneous activation potentials (SAPs) through electromyographic (EMG) recordings. EMG analysis documents the higher vulnerability of fast-fatigable MNs in the tibialis anterior (TA, a muscle mainly innervated by fast-fatigable MNs) when compared to slow-resistant MNs (more represented in the soleus, SOL). Starting at 10 weeks of age, a significant number of SAPs was detected in G93A TA muscles compared to WT mice. GCN2ib treatment delayed the appearance of SAP in TA muscles of G93A mice, which only became significant at 14 weeks. A similar trend was observed in SOL recordings from G93A mice. No effect in EMG recordings was found in GCN2ib-treated WT mice (Fig. 1i, j) (Additional file 1: Supplementary Statistical Analysis in Fig. 1j). Accordingly, GCN2ib treatment delayed the clinical score and the motor phenotype of G93A mice until 14 weeks old (Fig. 1k, l; Fig. S10). Moreover, GCN2ib treatment increased the weight gain of both WT and G93A mice (Fig. 1m; Fig. S10). To evaluate if GCN2 inhibition enhanced survival of spinal cord MNs, choline acetyltransferase-positive (ChAT+) neurons (MNs) were analyzed in independent WT and G93A mouse littermates treated and sacrificed at 12 weeks of age. In GCN2ib-treated G93A mice the number of MNs tended to be higher than that in vehicle-treated G93A mice (Fig. S11). Altogether, these results indicate that GCN2 pharmacological inhibition delays disease progression in ALS mice.

Our findings support the notion that stress-induced ISR contributes to the neurotoxicity of mutant SOD1 proteins. Indeed, ISR activation facilitates ALS neurotoxicity in other fALS experimental models. In the case of pathological (GGGGCC) expansions in the *C9ORF72* intron, repeat-associated non-AUG translation of dipeptide repeats (DpR) is enhanced by ISR activation [7]; in turn, DpR peptides trigger the ISR. In the case of TDP43 or FUS fALS, ISR-induced SGs act as “seeds” for protein aggregation, promoting cytosolic toxicity and/or nuclear loss-of-function neurotoxicity [6]. Similarly, our work demonstrates that ISR inhibition changes the behavior/toxicity of fALS neurotoxic proteins, dampening the initial trigger for motoneuron death.

Remarkably, PERK inhibition promotes WT (but not mutant) SOD1 clustering. Tampering with basal or ALS-induced PERK activity may alter ER homeostasis, affecting WT SOD1 redox regulation and promoting its aggregation [8]. However, PERK inhibition [5] cannot improve the survival of mutant SOD1-expressing neurons and fails to improve disease progression in transgenic mutant SOD1 mice [9], indicating that PERK is not the main/sole driver of ISR in ALS [8].

GCN2 is activated by uncharged tRNAs or by ribosome stalling. As a plausible trigger of these stresses, low amino acid levels have been documented in serum and cerebrospinal fluid of ALS patients. Moreover, glucose hypometabolism induces GCN2 activation and neurotoxicity in a C9ORF72 model [10], suggesting that GCN2 inhibition could be therapeutic for different ALS subtypes.

The discovery of GCN2 as a key determinant of mutant SOD1 behavior and neurotoxicity opens up a new perspective, where understanding how ISR determines proteostasis, and exploring GCN2 neuroprotective effect in other ALS models will serve to harness its therapeutic potential.

## Supplementary Information

**Additional file 1.** **Figure S1.** Overexpression of mutant (G93A, G85R) and WT SOD1 induces foci formation in the cytoplasm of HEK293 cells. **Figure S2.** A semi-automated image analysis tool (FociCount) to identify and analyze SOD1 foci formation. **Figure S3**. ISRIB-mediated ISR modulation reduces the percentage of HEK293 cells forming SOD1 cytoplasmic foci. **Figure S4**. Pharmacological and genetic strategies for ISR kinase modulation. **Figure S5.** Effect of HRI, PKR, PERK, and GCN2 knock-down in the intracellular distribution of WT SOD1. **Figure S6.** Effect of HRI, PKR, PERK, and GCN2 knock-down in the intracellular distribution of mutant SOD1 (G93A). **Figure S7. **Effect of PERK and GCN2 pharmacological inhibition in the intracellular distribution of SOD1 (WT and mutant G93A). **Figure S8.** PERK pharmacological inhibition determines the distribution of WT SOD1 in primary neurons. **Figure S9**. Characterization of the effect of genetic and pharmacological GCN2 inhibition in the survival of mCherry-expressing neurons. **Figure S10.** Effect of GCN2ib in symptomatic progression, strength and body weight in ALS and WT mice. **Figure S11.** GCN2ib treatment of SOD1^G93A^ transgenic mice (G93A) delays the death of spinal cord ChAT^+^ motoneurons. **Table S1.** Cox Proportional Hazard analysis of the effect of CRISPR-Cas9 mediated GCN2 inhibition in neuronal survival. **Table S2.** Cox Proportional Hazard analysis of the effect of pharmacological GCN2 inhibition with A92 in neuronal survival. **Supplementary Statistical Analysis.**

**Additional file 2**. **Materials and Methods**.

## Data Availability

The datasets used and/or analyzed during the current study are available from the corresponding author on reasonable request.

## Abbreviations

FUS: Fused in sarcoma
GCN2: general control nonderepressible 2
HRI: heme-regulated inhibitor
iPSC: Induced pluripotent stem cells
PERK: Protein kinase RNA-like endoplasmic reticulum kinase
PKR: protein kinase R
SOD1: superoxide dismutase type 1
TDP43: TAR DNA-binding protein 43
