# Supplementary material for: GCN2 inhibition reduces mutant SOD1 clustering and toxicity and delays disease progression in an amyotrophic lateral sclerosis mouse model
Source: Transl Neurodegener. 2024 Sep 20;13:49. doi: 10.1186/s40035-024-00441-w (PMC11414287; doi:10.1186/s40035-024-00441-w)
Supplement: Supplementary file 1 — Additional file 1. Figure S1. Overexpression of mutant (G93A, G85R) and WT SOD1 induces foci formation in the cytoplasm of HEK293 cells. Figure S2. A semi-automated image analysis tool (FociCount) to identify and analyze SOD1 foci formation. Figure S3. ISRIB-mediated ISR modulation reduces the percentage of HEK293 cells forming SOD1 cytoplasmic foci. Figure S4. Pharmacological and genetic strategies for ISR kinase modulation. Figure S5. Effect of HRI, PKR, PERK, and GCN2 knock-down in the intracellular distribution of WT SOD1. Figure S6. Effect of HRI, PKR, PERK, and GCN2 knock-down in the intracellular distribution of mutant SOD1 (G93A). Figure S7. Effect of PERK and GCN2 pharmacological inhibition in the intracellular distribution of SOD1 (WT and mutant G93A). Figure S8. PERK pharmacological inhibition determines the distribution of WT SOD1 in primary neurons. Figure S9. Characterization of the effect of genetic and pharmacological GCN2 inhibition in the survival of mCherry-expressing neurons. Figure S10. Effect of GCN2ib in symptomatic progression, strength and body weight in ALS and WT mice. Figure S11. GCN2ib treatment of SOD1G93A transgenic mice (G93A) delays the death of spinal cord ChAT+ motoneurons. Table S1. Cox Proportional Hazard analysis of the effect of CRISPR-Cas9 mediated GCN2 inhibition in neuronal survival. Table S2. Cox Proportional Hazard analysis of the effect of pharmacological GCN2 inhibition with A92 in neuronal survival. Supplementary Statistical Analysis. [file 40035_2024_441_MOESM1_ESM.docx]

## Additional file 1.


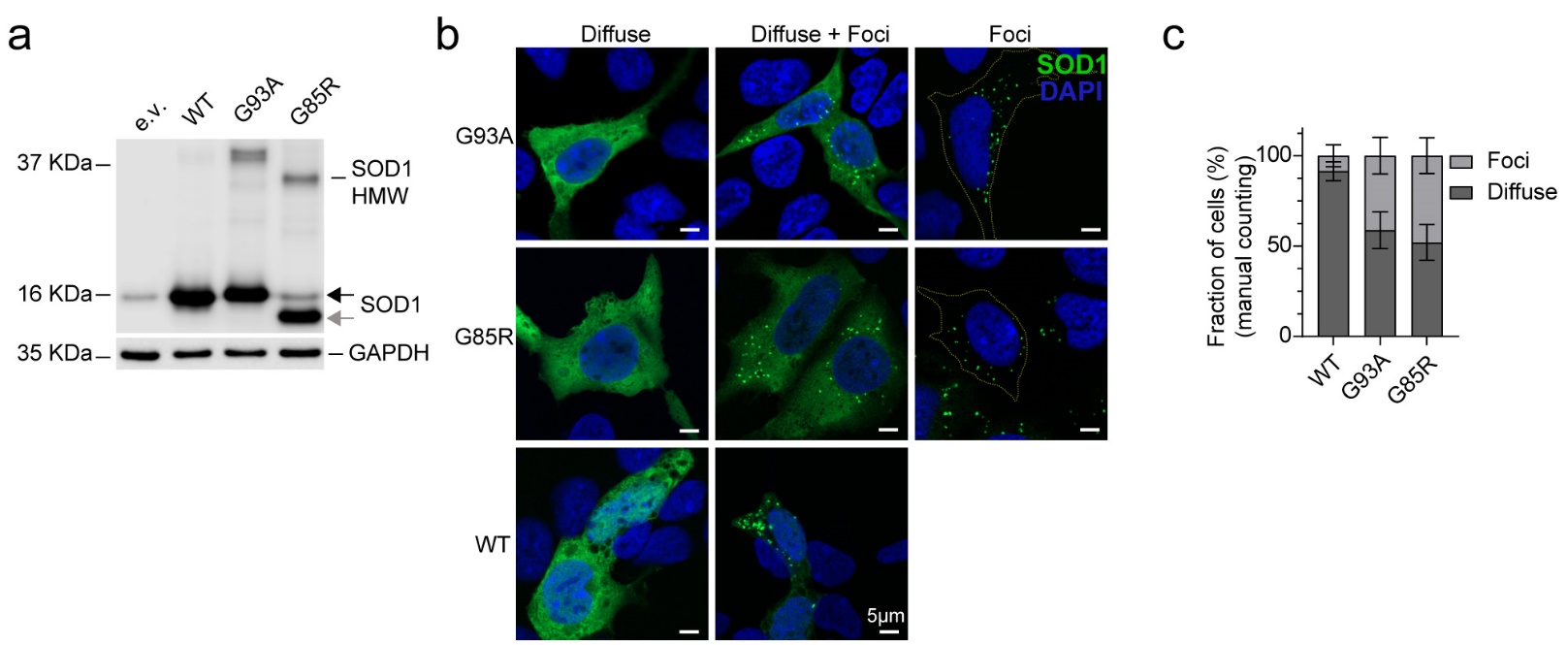


**Figure S1.** Overexpression of mutant (G93A, G85R) and WT SOD1 induces foci formation in the cytoplasm of HEK293 cells. **a** WB analysis of protein extracts from HEK293 cells transiently transfected with WT and mutant (G93A and G85R) SOD1 variants (GAPDH as loading control). HMW, high molecular weight; e.v., empty vector. The grey arrow shows mutant G85R SOD1. **b** Representative images of the SOD1 distribution pattern in HEK293 cells transiently transfected with plasmids expressing mutant SOD1 (G93A, G85R) or WT versions. Ninety-six hours post-transfection, cells were analyzed by immunofluorescence with a specific anti-SOD1 antibody. SOD1 pattern distribution is observed either as diffuse throughout the entire cytoplasm, diffuse with the presence of foci, or predominantly concentrated in foci. **c** Graph representation of the percentage of cells (%) with SOD1 diffuse or forming foci on each condition (WT, G93A, G85R) (manual counting). Mean +/- SD.

**
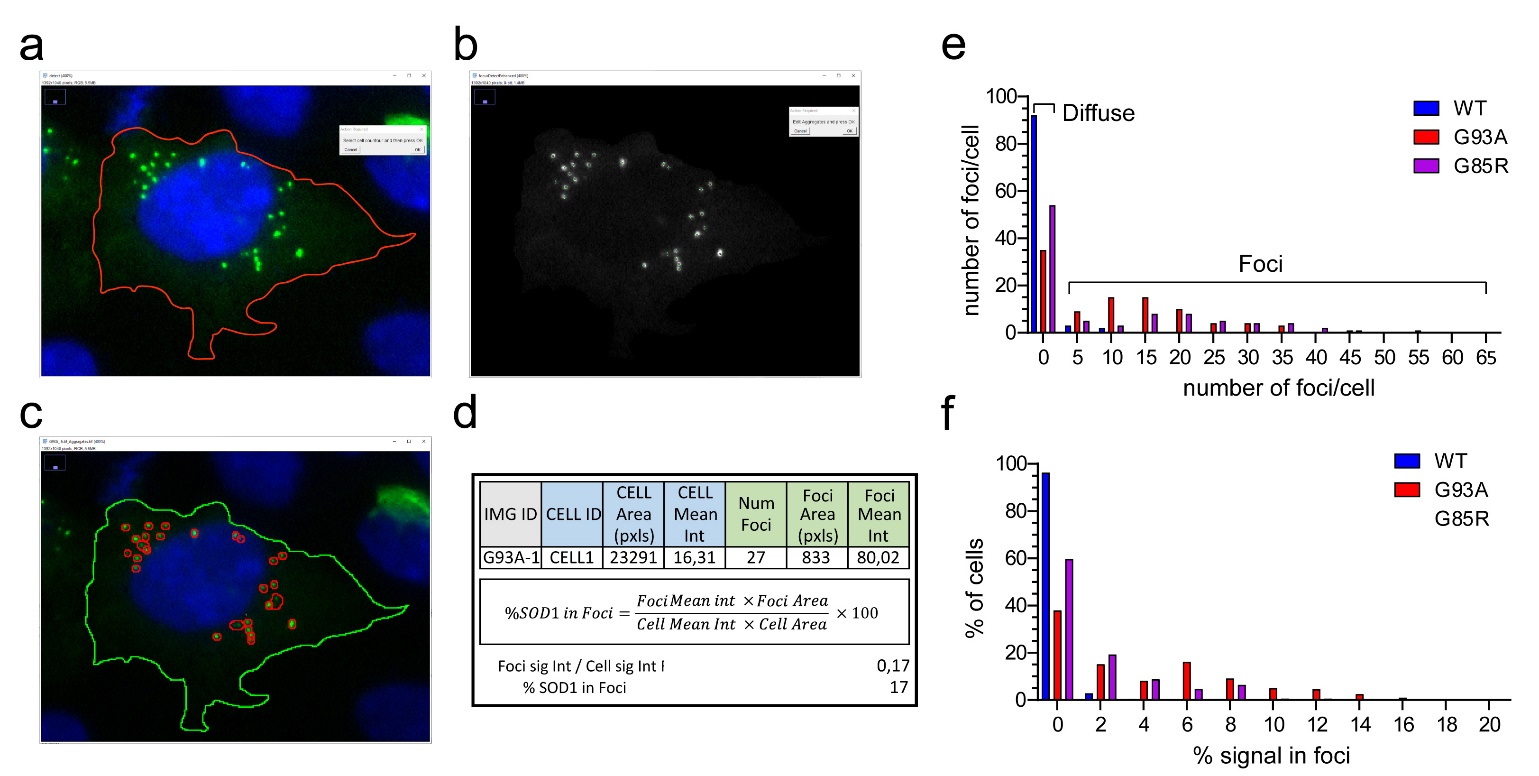
**

**Figure S2.** A semi-automated image analysis tool (FociCount) to identify and analyze SOD1 foci formation. To score foci formation in a quantitative and unbiased way, we developed an image analysis tool (FociCount). FociCount facilitates the determination of the cellular contour and identifies SOD1 foci in a semi-automated manner, reporting the number of foci per cell and the fraction of intracellular SOD1 concentrated into foci. **a-c** Representative images of the image processing. **d** Representative output of FociCount analysis of HEK293 cells expressing G93A mutant SOD1. **e, f** Representative experiment where the clustering of WT, G93A, and G85R proteins was characterized (out of three independent experiments performed). As it can be observed, the number of foci per cell is variable. Thus, in most experiments, we quantified the fraction of cells containing at least one SOD1 focus. **e** Frequency distribution graph depicting the number of SOD1 foci per cell. **f** Frequency distribution graph depicting the fraction of SOD1 signal in foci. A minimum of 200 cells were analyzed for each condition. Even in those cells where all fluorescence signal was apparently concentrated into foci, focal signal never exceeded 10% of the total cellular signal.


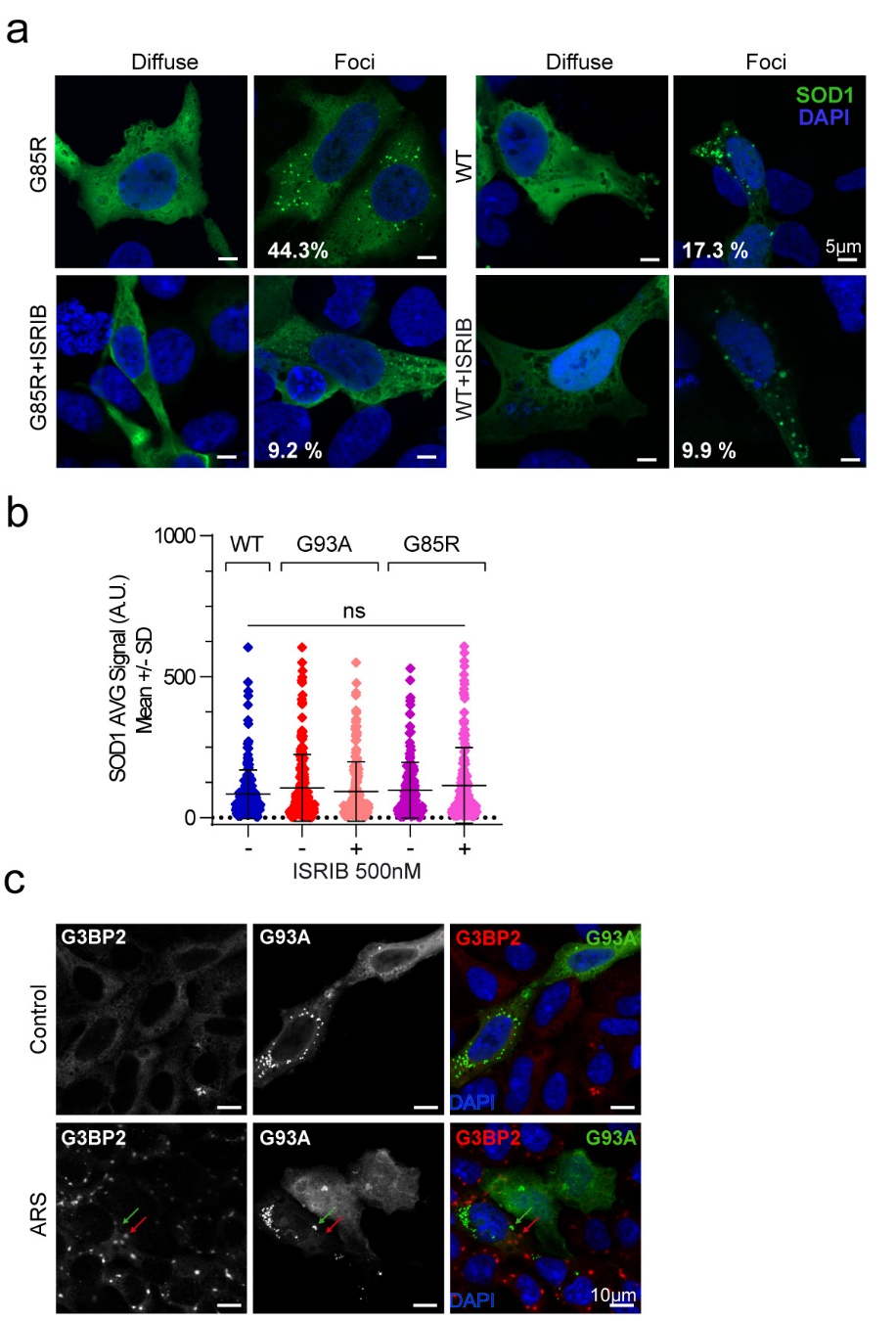


**Figure S3.** ISRIB-mediated ISR modulation reduces the percentage of HEK293 cells forming SOD1 cytoplasmic foci. **a** Images of a representative experiment showing SOD1 distribution patterns (diffuse and in foci) in HEK293 cells transiently transfected with mutant (G85R) or wild-type SOD1 (WT) +/- ISRIB 500nM treatment. Immunofluorescence with anti-SOD1 antibody. The number in each picture indicates the percentage (%) of cells with foci. In the few cells where SOD1 still clustered into foci after ISRIB treatment, no significant changes in the number or size/shape of the foci were observed. **b** Graph showing the average (AVG) fluorescence intensity values measured in whole (nucleus and cytoplasm) SOD1-positive cells from each condition. FociCount analysis, data from *n=*3 experiments with ≥100 cells/condition on each experiment, Kruskal-Wallis test. **c** Representative images of HEK293 cells transiently transfected with mutant SOD1 (G93A) +/- ARS (sodium arsenite) to induce stress granules (SGs). IF experiments with anti-G3BP2 antibody (a marker of SGs). Mutant SOD1 overexpression does not induce the assembly of SG. This result could be anticipated because the mild ISR activation induced by mutant SOD1 (Bugallo R. et al., 2020, Cell Death and Disease) should not be sufficient to promote SG formation. Still, we asked if mutant SOD1 could be recruited to SGs structures formed after treatment with the robust ISR inducer sodium arsenite (ARS). Even under these forced conditions, mutant SOD1 foci failed to colocalize with SGs. Red and green arrows indicate SG and SOD1 foci, respectively. A.U., arbitrary units; SD, standard deviation; ns, non-statistically significant difference; ****P<*0.001.


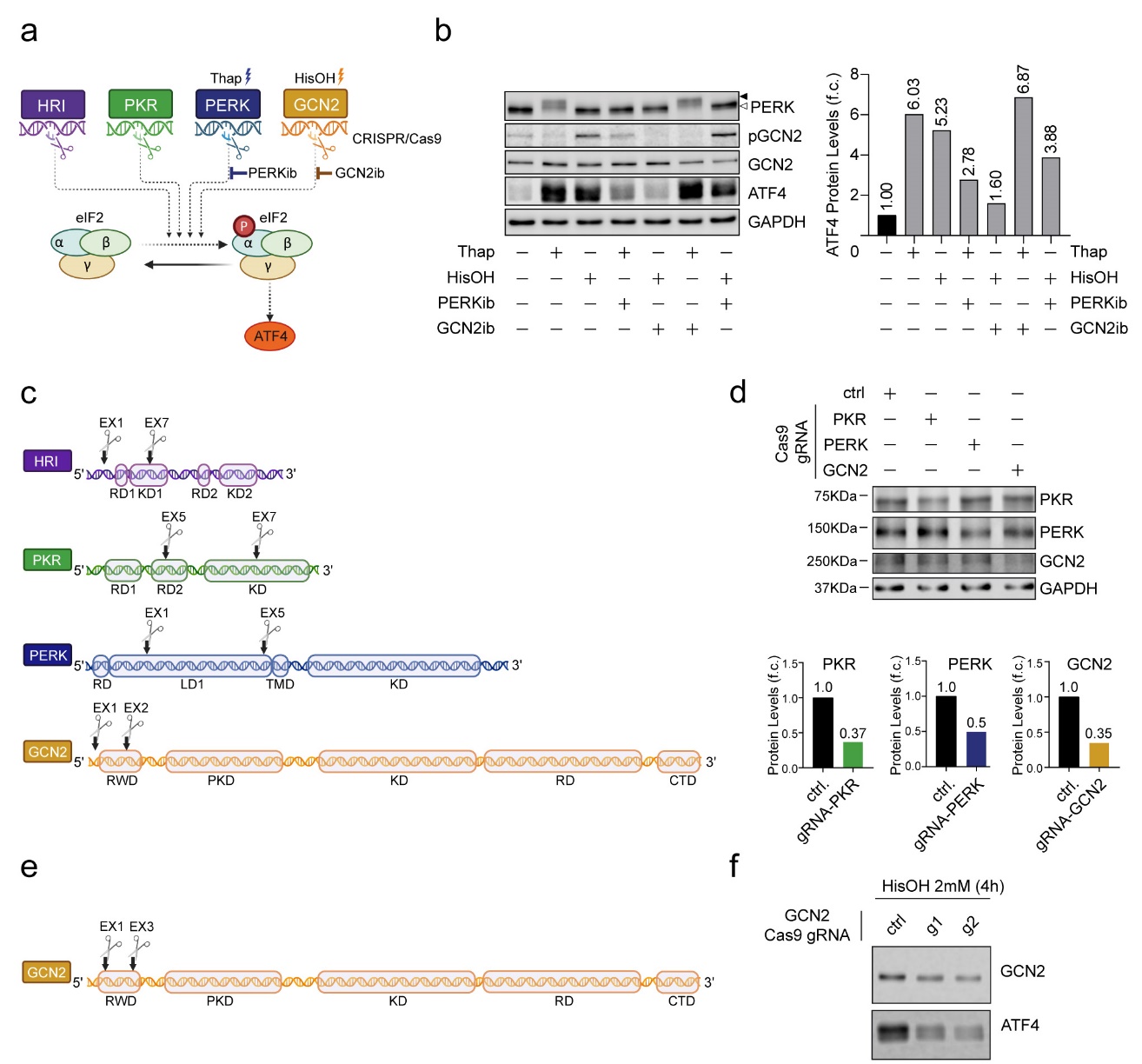


**Figure S4.** Pharmacological and genetic strategies for ISR kinase modulation. **a** Diagram illustrating ISR signaling pathways and outcomes of PERK and GCN2 genetic (CRISPR/Cas9) and pharmacological inhibition under Thapsigargin (Thap) or Histidinol (HisOH) stress induction; PERKib (PERK inhibitor GSK2606414), GCN2ib (GCN2 inhibitor). **b** Evaluation of pharmacological PERK and GCN2 inhibition with PERKib and GCN2ib. Western-blot analysis of protein extracts from HEK293 cells treated with Thap (200nM) +/- PERKib (500nM) or HisOH 2 mM +/- GCN2ib (1µM) revealed an effective and specific Thap-dependent PERK activation (PERK phosphorylation, P-PERK) and HisOH-dependent GCN2 activation (GCN2 phosphorylation, P-GCN2) as well as ATF4 translation. PERKib and GCN2ib prevent PERK and GCN2 phosphorylation and, consequently, ATF4 translation. Quantification of ATF4 protein levels at each condition is shown. Design and efficacy of CRISPR/Cas9 guides to knock down human and rat ISR kinases (PERK, PKR, HRI, and GCN2). **c** Scheme representing DNA domains of human ISR kinase genes (HRI, PKR, PERK, and GCN2) and location of Crispr/cas9-dependent insertions or deletions (represented with scissors). **d** Western-blot analysis of protein extracts from HEK293 cells transfected with pX458 plasmids expressing human Crispr/cas9 guides (CRISPR-PKR, CRISPR-PERK, CRISPR-GCN2) and pX458 empty vector (control). Human CRISPR-PKR, CRISPR-PERK, and CRISPR-GCN2 guides decrease PKR, PERK, and GCN2 protein levels, respectively. Quantification of PKR, PERK, and GCN2 protein levels is shown.  **e** Scheme representing DNA domains of rat GCN2 gene and location of Crispr/cas9-dependent insertions or deletions. **f** Western-blot analysis of protein extracts from RH7777 cells transfected with pX458 plasmids expressing GCN2 Crispr/cas9 guides (G1, G2, G3) and pX458 empty vector (control) and treated with +/- Histidinol 2 mM (GCN2 activator) for 4 hours (4h). GCN2 and ATF4 protein levels decrease in cells expressing GCN2 Crispr/cas9 guides.


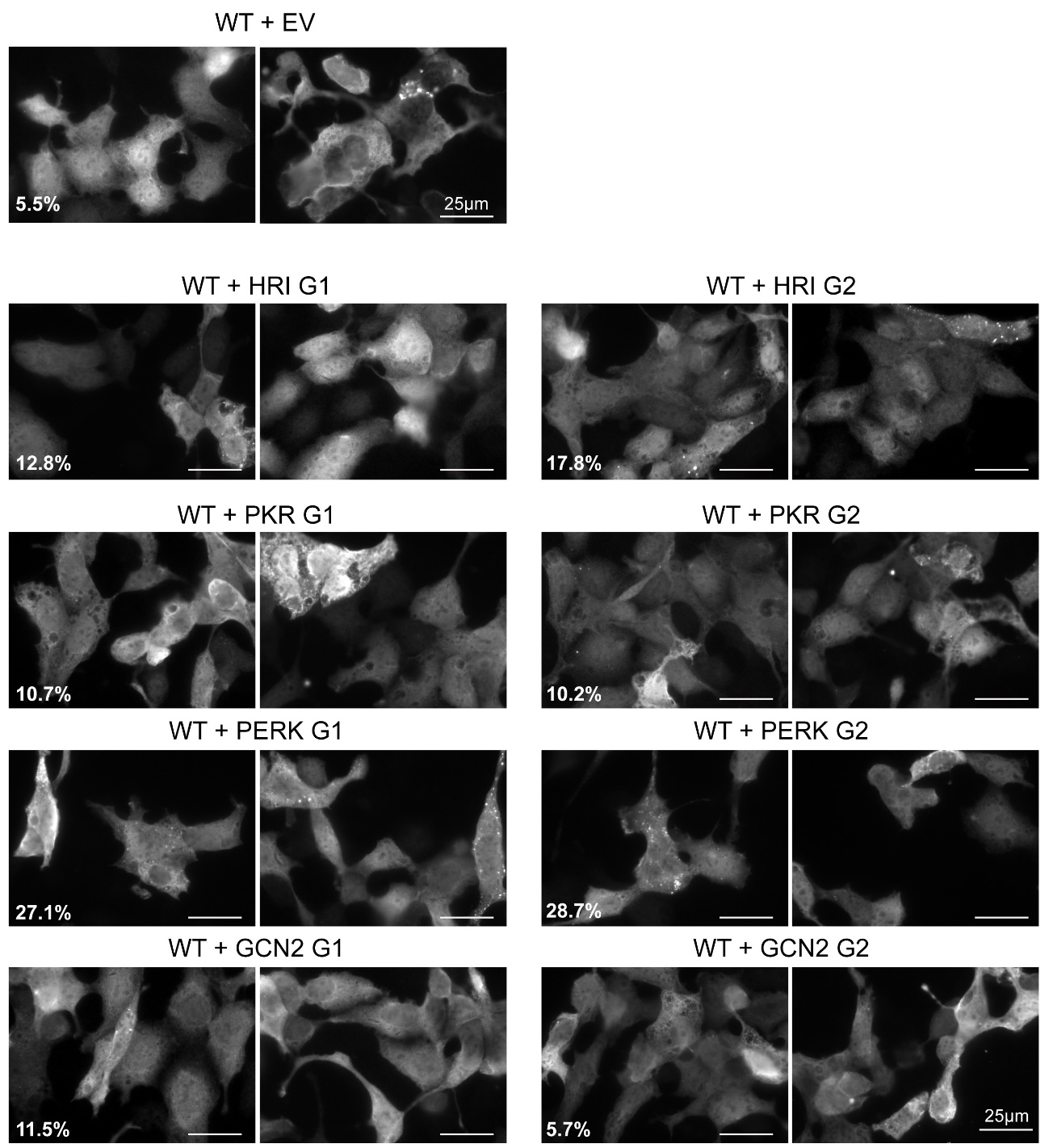


**Figure S5.** Effect of HRI, PKR, PERK, and GCN2 knock-down in the intracellular distribution of WT SOD1. Representative images from experiments quantified in Fig1B of HEK293 cells co-transfected with plasmids expressing wild-type SOD1 (WT) and pX458 plasmids expressing Cas9 endonuclease with two independent gRNAs (G1, G2) targeting HRI, PKR, PERK and GCN2 genes or pX458 expressing a non-targeting gRNA (EV). Immunofluorescences with anti-SOD1 antibody showing SOD1 distribution patterns (diffuse and in foci). Numbers indicate percentage (%) of cells with foci.


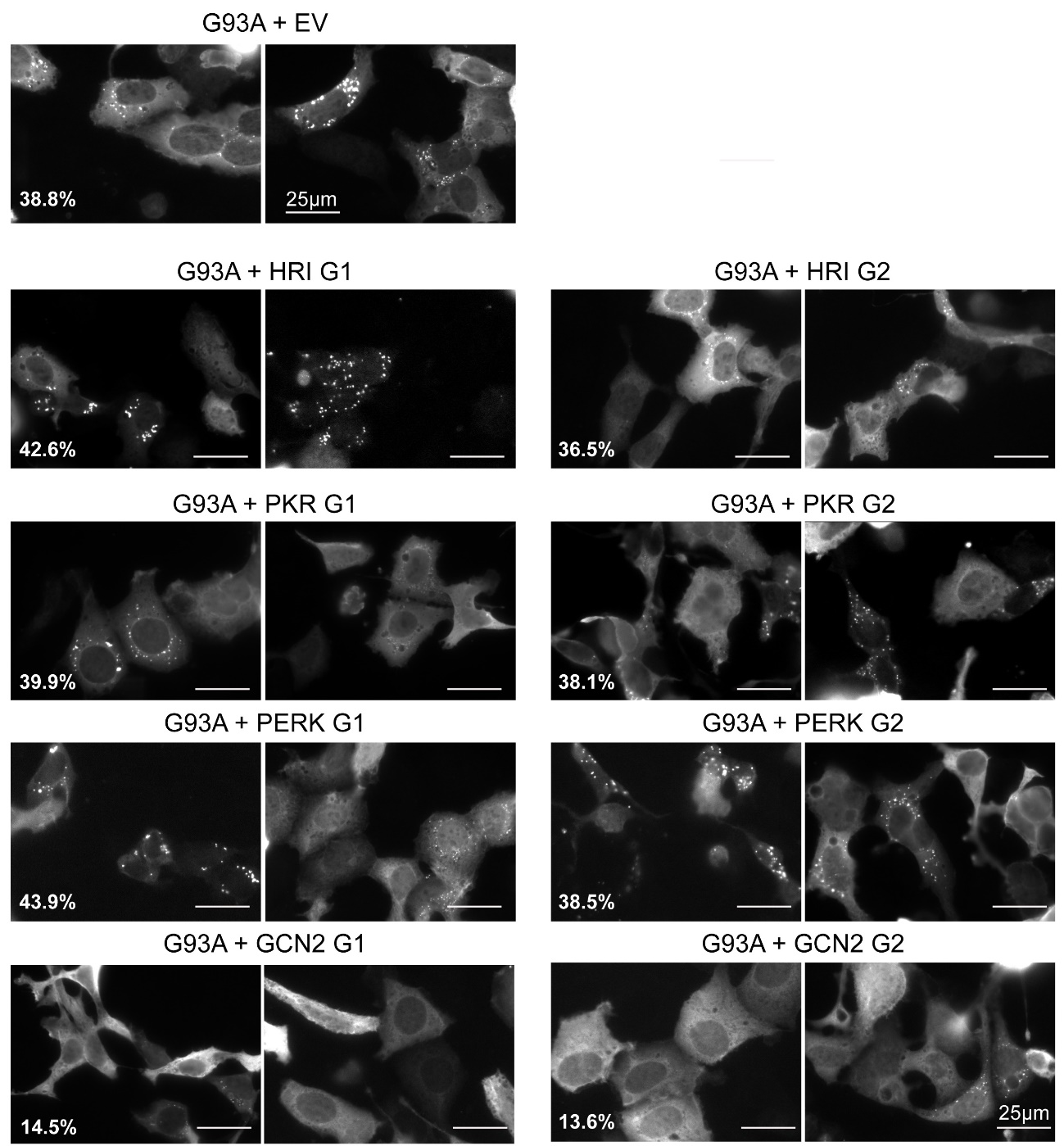


**Figure S6.** Effect of HRI, PKR, PERK, and GCN2 knock-down in the intracellular distribution of mutant SOD1 (G93A). Representative images from experiments quantified in Fig1B of HEK293 cells co-transfected with plasmids expressing mutant SOD1 (G93A) and pX458 plasmids expressing Cas9 endonuclease with two independent gRNAs (G1, G2) targeting HRI, PKR, PERK and GCN2 genes or pX458 expressing a non-targeting gRNA (EV). Immunofluorescences with anti-SOD1 antibody showing SOD1 distribution patterns (diffuse and in foci). Numbers indicate percentage (%) of cells with foci.


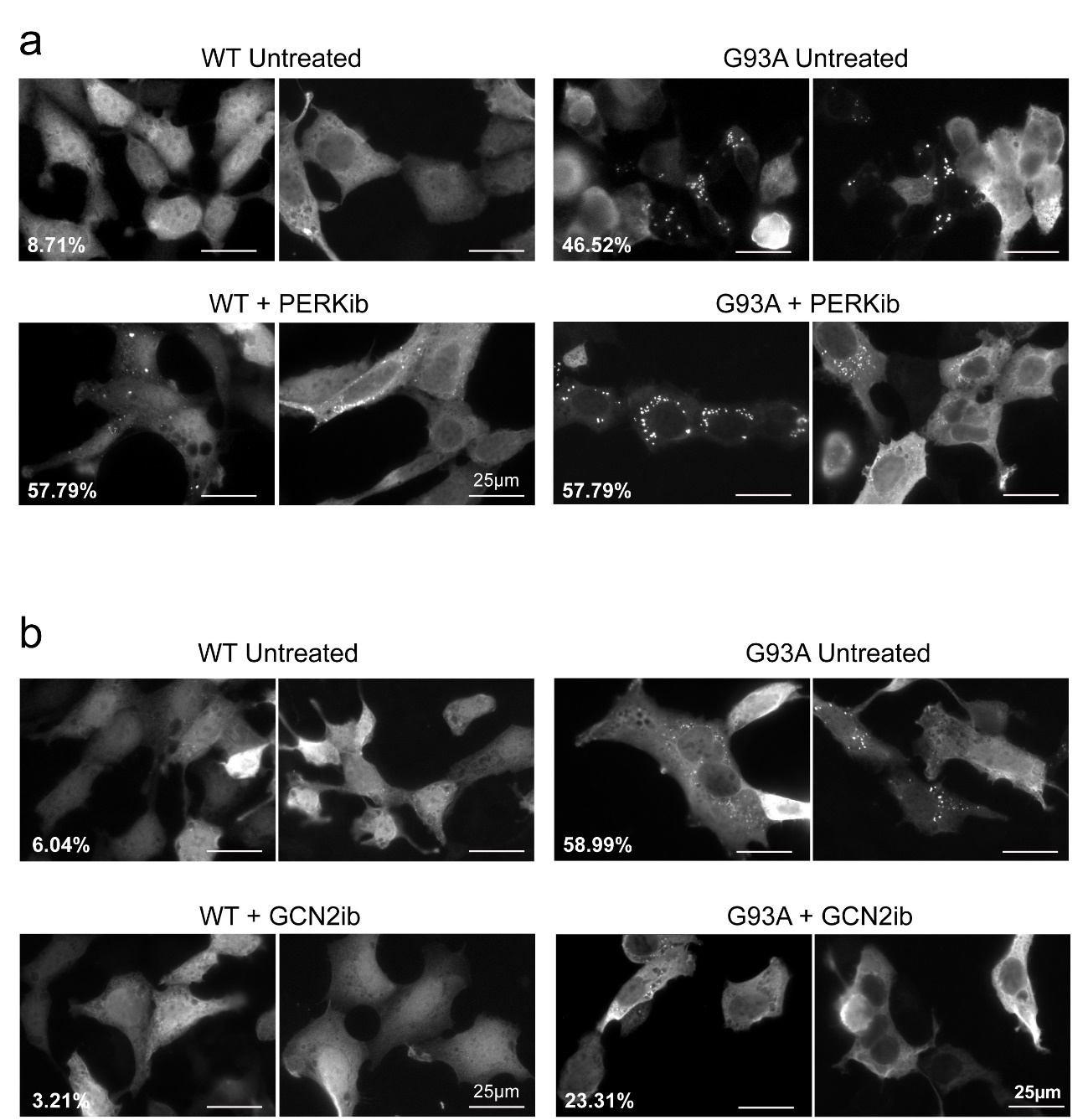


**Figure S7.** Effect of PERK and GCN2 pharmacological inhibition in the intracellular distribution of SOD1 (WT and mutant G93A). Representative images from experiments quantified in Fig1C-D of HEK293 cells transfected with plasmids expressing SOD1 (WT and mutant G93A) treated or untreated with PERK or GCN2 pharmacological inhibitors (PERKib 500nM or GCN2ib 1μM). Immunofluorescences with anti-SOD1 antibody showing SOD1 distribution patterns (diffuse and in foci). Numbers indicate percentage (%) of cells with foci.


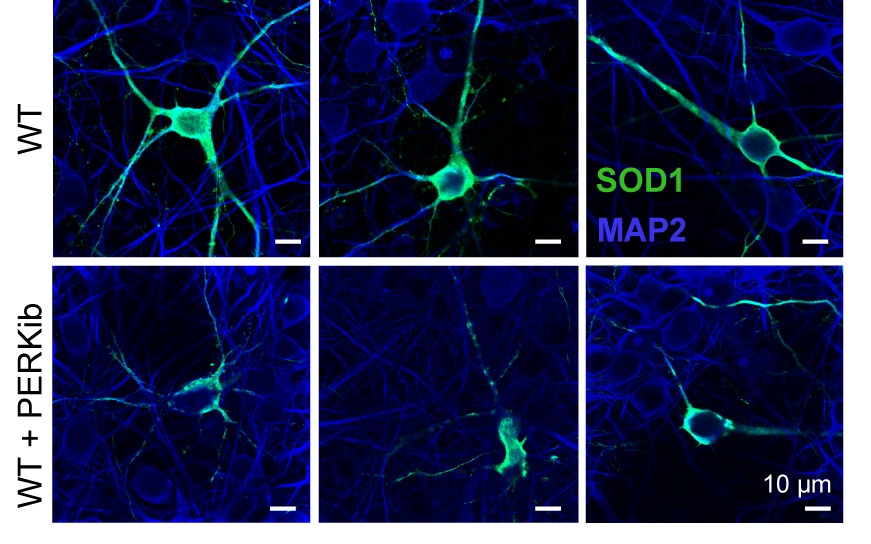


**Figure S8.** PERK pharmacological inhibition determines the distribution of WT SOD1 in primary neurons. Representative confocal images of immunofluorescences experiments illustrating SOD1 intraneuronal distribution (green) in primary neurons expressing WT SOD1 -/+ PERKib. In blue, MAP2 staining identifies neurons. PERK inhibition increases clustered WT SOD1 distribution.

**
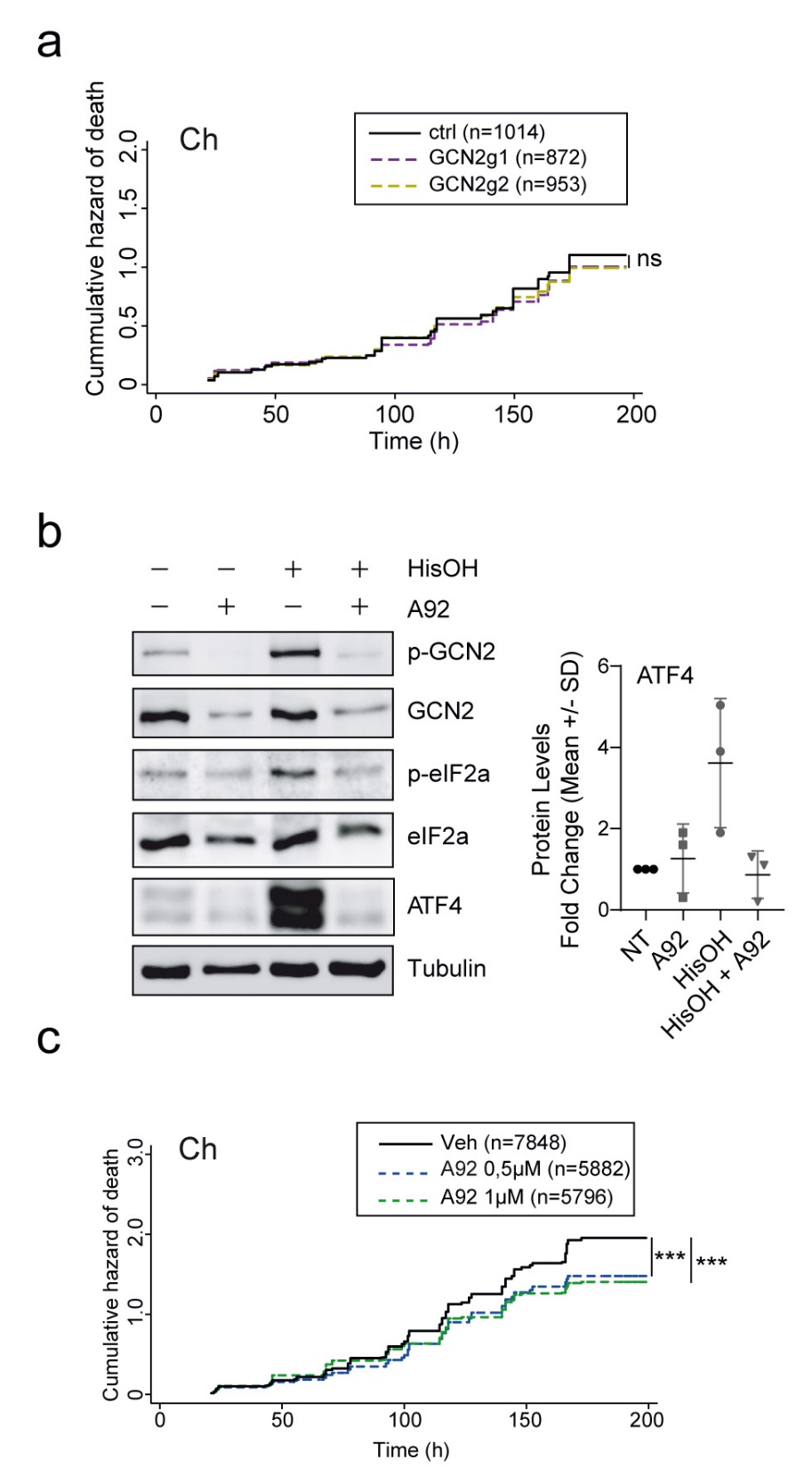
**

**Figure S9**. Characterization of the effect of genetic and pharmacological GCN2 inhibition in the survival of mCherry-expressing neurons**. a** Cumulative death hazard estimation of primary neurons co-transfected with mCherry (Ch) and sgRNAs-containing CRISPR/Cas9 plasmids for GCN2 targeting (GCN2g1, GCN2g2) or px458 empty vector (control (ctrl)). Cox Proportional Hazard (CPH) analysis; pooled data from three independent experiments (Table S1). **b** A92 inhibits GCN2 activation (phosphorylation_pGCN2) and signaling events (ATF4 translation) in Histidinol (His OH)-treated HEK293 cells. Representative WB analysis and quantification of ATF4 protein levels on each condition in *n=*3 independent experiments. **c** Cumulative death hazard estimation of primary neurons transfected with Ch and treated with GCN2 pharmacological inhibitor (A92) or vehicle (DMSO). Cox Proportional Hazard analysis; pooled data from five independent experiments (Table S2). Number of neurons (n); ns, not statistically different; ****P* < 0.001.


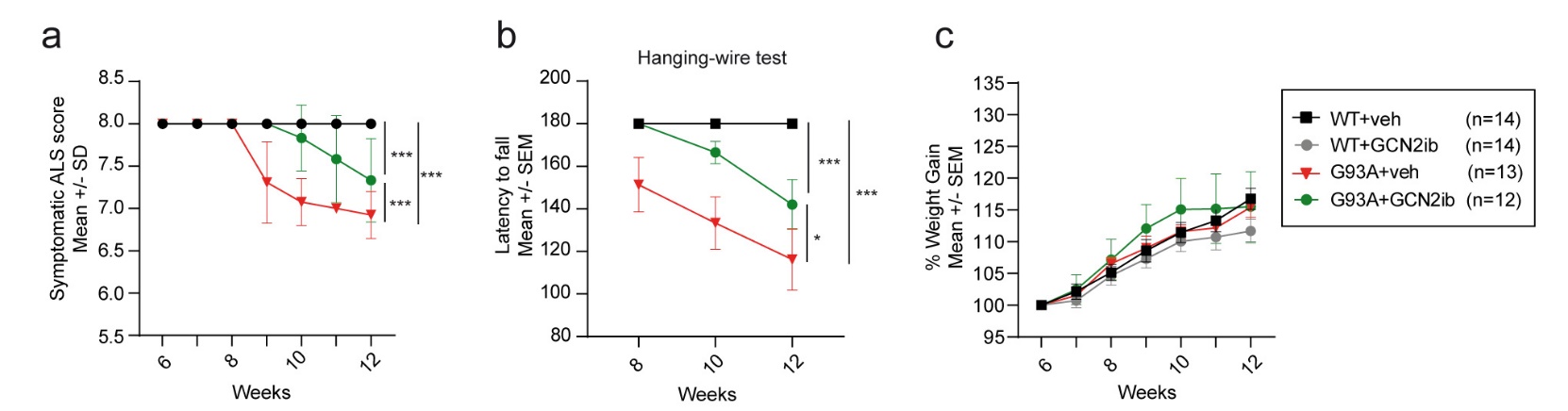


**Figure S10**. Effect of GCN2ib in symptomatic progression, strength and body weight in ALS and WT mice. Results from two additional independent experiments were performed in which male mice were treated with GCN2ib from 6 to 12 weeks-old as indicated in Figure 1**.** Data represents pool results from ExpA (WT n=5, WT+GCN2ib n=5, G93A n=4, G93A+GCN2ib n=5) and ExpB (WT n=9, WT+GCN2ib n=9, G93A n=9, G93A+GCN2ib n=7). **a** Clinical score, **b** Motor phenotype and **c** weight gain. Two-way repeated measures ANOVA and Tukey´s post-hoc test. **P* < 0.05; ****P* < 0.001.

**
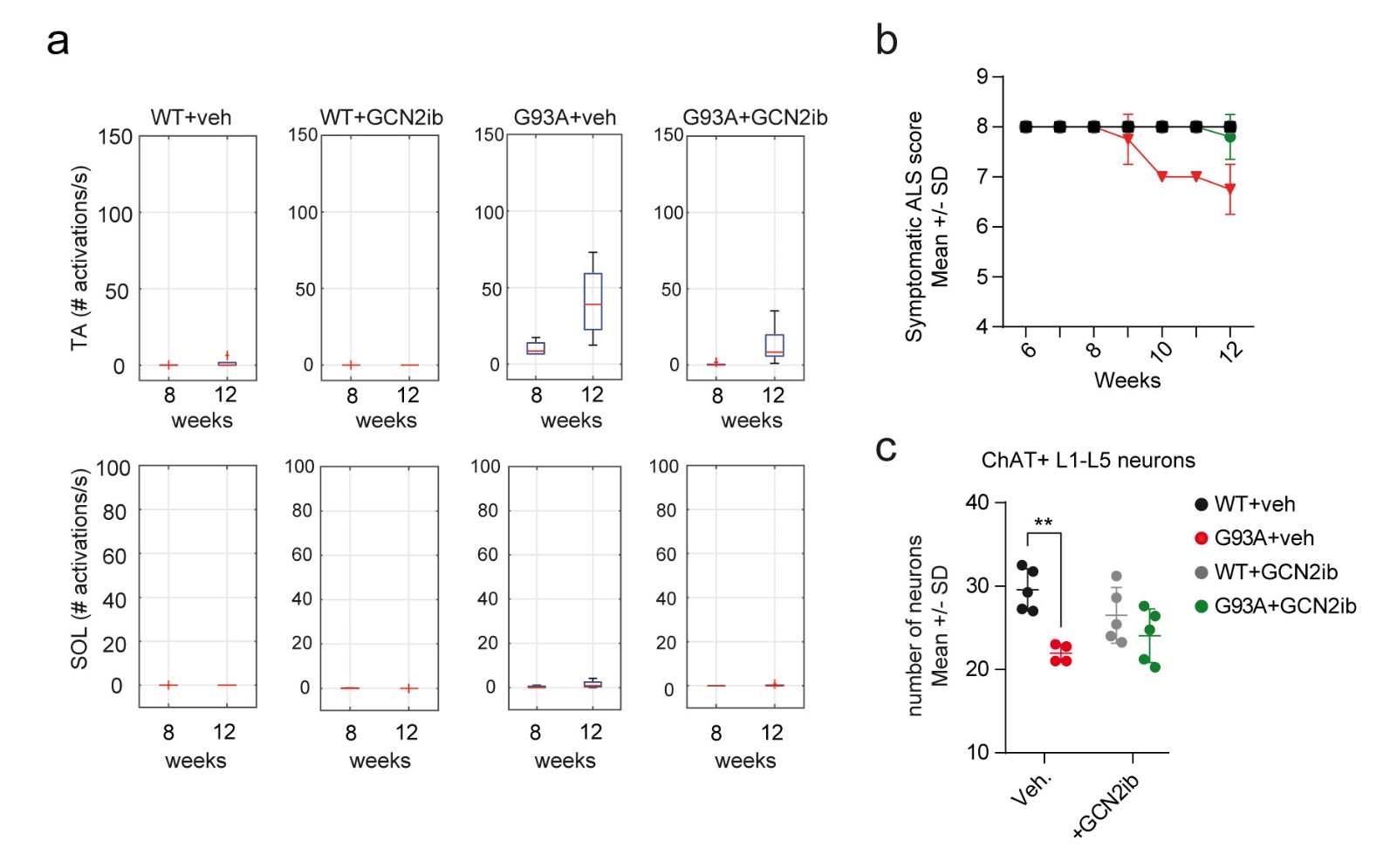
**

**Figure S11.** GCN2ib treatment of SOD1^G93A^ transgenic mice (G93A) delays the death of spinal cord ChAT+ motoneurons. A cohort of animals (ExpA) was treated with GCN2ib from 6 weeks old (as in Fig. 1) but sacrificed at 12 weeks (WT *n=*5, WT+GCN2ib *n=*5, G93A *n=*4, G93A+GCN2ib *n=*5). **a** Longitudinal EMG analysis in TA and SOL from 8 to 12 weeks from WT and G93A mice +/-GCN2ib. As already observed in Figure 1, GCN2ib-treated G93A mice experience a delay in the number of activations per second (# activations/s) compared to non-treated G93A mice. Two-way Repeated Measures (RM) ANOVA; Time (F_1, 7_ = 9.966, *P<*0.05), Condition (F_1, 7_ = 7.687, *P<* 0.05). Box plots with median and 25th and 75th percentile (“+” outlier). **b** GCN2ib treatment delays the disease clinical score of non-treated G93A mice. Two-way RM ANOVA; Time (F_6,42_ = 16.82, *P<*0.001), Condition (F_1, 7_ = 94.11, *P<* 0.05). **c** Quantification of ChAT + motoneurons at 12 weeks in WT and G93A +/-GCN2ib treatment in parallel mice cohorts. Two-way ANOVA (Condition F_1,15_ = 15.58, ***P* < 0.01); (Interaction F_1,15_ = 4.101, p =0.06). SD: standard deviation.

**Table S1.** Cox Proportional Hazard analysis of the effect of CRISPR-Cas9 mediated GCN2 inhibition in neuronal survival.

| Reference  (HR=1) | Groups | HR | Robust Std. Err. | *P*-value | CI |
| --- | --- | --- | --- | --- | --- |
| G93ACh (ctrl) | G93ACh_GCN2g1 | 0,76 | 0,049 | < 0,001*** | 0,67 - 0,87 |
| G93ACh (ctrl) | G93ACh_GCN2g2 | 0,78 | 0,037 | < 0,001*** | 0,70 - 0,85 |
| Ch (ctrl) | Ch_GCN2g1 | 0,97 | 0,077 | 0,696 | 0,83 - 1,13 |
| Ch (ctrl) | Ch_GCN2g2 | 0,99 | 0,077 | 0,971 | 0,85 - 1,16 |

Hazard ratio (HR) estimation for each group considering G93ACh (ctrl) or Ch (ctrl) as references (HR=1). Data from three independent experiments per condition (6 total plates). Robust standard error (Robust Std. Err.) adjusted by clusters (wells) and data stratified by plate. Control (ctrl), 95% CI (Confidence Interval).

**Table S2.** Cox Proportional Hazard analysis of the effect of pharmacological GCN2 inhibition with A92 in neuronal survival.

| Reference  (HR=1) | Groups | HR | Robust Std. Err. | *P*-value | CI |
| --- | --- | --- | --- | --- | --- |
| G93ACh (veh) | G93ACh+A92 (0,5µm) | 0,71 | 0,0225 | < 0,001*** | 0,67 - 0,76 |
| G93ACh (veh) | G93ACh+A92 (1µm) | 0,82 | 0,0257 | < 0,001*** | 0,77 - 0,87 |
| Ch (veh) | Ch+A92 (0,5µm) | 0,78 | 0,026 | < 0,001*** | 0,73 - 0,84 |
| Ch (veh) | Ch+A92 (1µm) | 0,86 | 0,028 | < 0,001*** | 0,80 - 0,91 |

Hazard ratio (HR) estimation for each group considering G93Ach (veh) or Ch (veh) as references (HR=1). Data from five independent experiments per condition (10 total plates). Robust standard error (Robust Std. Err.) adjusted by clusters (wells) and data stratified by plate. Vehicle (veh), 95% CI (Confidence Interval).

## Supplementary Statistical Analysis

**Supplementary Statistical Analysis in Figure 1j**

**EMG evolution in TA muscle**

**Repeated Measures ANOVA for 4 groups, 5 time points**

2-way mixed ANOVA comparing the evolution of EMG activity among the two genotypes and treatment (4 groups) reveals a significant effect of time, group, and interaction factors, probing that the evolution of the EMG activity is different in the different groups

| **Within Subjects Effects** | | | | | | | | | | | | | |
| --- | --- | --- | --- | --- | --- | --- | --- | --- | --- | --- | --- | --- | --- |
| **Cases** | | **Sphericity Correction** | | **Sum of Squares** | | **df** | | **Mean Square** | | ***F*** | | ***P*** | |
| Weeks |  | None |  | 11086.453 |  | 4.000 |  | 2771.613 |  | 9.120 |  | < .001 |  |
| Weeks ✻ Group |  | None |  | 15174.190 |  | 12.000 |  | 1264.516 |  | 4.161 |  | < .001 |  |
| Residuals |  | None |  | 21881.490 |  | 72.000 |  | 303.910 |  |  |  |  |  |
|  | | | | | | | | | | | | | |
| *Note.*  Type II Sum of Squares | | | | | | | | | | | | | |

| **Between Subjects Effects** | | | | | | | | | | | |
| --- | --- | --- | --- | --- | --- | --- | --- | --- | --- | --- | --- |
| **Cases** | | **Sum of Squares** | | **df** | | **Mean Square** | | ***F*** | | ***P*** | |
| Group |  | 17900.614 |  | 3 |  | 5966.871 |  | 10.058 |  | < .001 |  |
| Residuals |  | 10678.239 |  | 18 |  | 593.235 |  |  |  |  |  |
|  | | | | | | | | | | | |
| *Note.*  Type II Sum of Squares | | | | | | | | | | | |

| **Descriptives** | | | | | | | | | | | | | |
| --- | --- | --- | --- | --- | --- | --- | --- | --- | --- | --- | --- | --- | --- |
| **Weeks** | | **Group** | | **N** | | **Mean** | | **SD** | | **SE** | | **Coefficient of variation** | |
| week 8 |  | TG_GCN2iB |  | 6 |  | 0.075 |  | 0.082 |  | 0.034 |  | 1.099 |  |
|  |  | TG_Veh |  | 6 |  | 0.390 |  | 0.636 |  | 0.260 |  | 1.629 |  |
|  |  | WT_GCN2iB |  | 6 |  | 0.240 |  | 0.366 |  | 0.149 |  | 1.525 |  |
|  |  | WT_Veh |  | 4 |  | 0.030 |  | 0.030 |  | 0.015 |  | 1.016 |  |
| week 10 |  | TG_GCN2iB |  | 6 |  | 4.946 |  | 3.845 |  | 1.570 |  | 0.778 |  |
|  |  | TG_Veh |  | 6 |  | 7.265 |  | 8.765 |  | 3.578 |  | 1.206 |  |
|  |  | WT_GCN2iB |  | 6 |  | 0.721 |  | 1.022 |  | 0.417 |  | 1.418 |  |
|  |  | WT_Veh |  | 4 |  | 0.759 |  | 1.164 |  | 0.582 |  | 1.535 |  |
| week 12 |  | TG_GCN2iB |  | 6 |  | 5.698 |  | 4.109 |  | 1.677 |  | 0.721 |  |
|  |  | TG_Veh |  | 6 |  | 42.076 |  | 23.830 |  | 9.728 |  | 0.566 |  |
|  |  | WT_GCN2iB |  | 6 |  | 0.140 |  | 0.230 |  | 0.094 |  | 1.643 |  |
|  |  | WT_Veh |  | 4 |  | 0.375 |  | 0.519 |  | 0.259 |  | 1.384 |  |
| week 14 |  | TG_GCN2iB |  | 6 |  | 28.947 |  | 16.925 |  | 6.910 |  | 0.585 |  |
|  |  | TG_Veh |  | 6 |  | 68.733 |  | 67.517 |  | 27.564 |  | 0.982 |  |
|  |  | WT_GCN2iB |  | 6 |  | 0.160 |  | 0.153 |  | 0.062 |  | 0.952 |  |
|  |  | WT_Veh |  | 4 |  | 0.179 |  | 0.326 |  | 0.163 |  | 1.819 |  |
| week 16 |  | TG_GCN2iB |  | 6 |  | 41.716 |  | 24.099 |  | 9.838 |  | 0.578 |  |
|  |  | TG_Veh |  | 6 |  | 35.382 |  | 20.177 |  | 8.237 |  | 0.570 |  |
|  |  | WT_GCN2iB |  | 6 |  | 0.069 |  | 0.093 |  | 0.038 |  | 1.345 |  |
|  |  | WT_Veh |  | 4 |  | 0.000 |  | 0.000 |  | 0.000 |  | NaN |  |
|  | | | | | | | | | | | | | |

**Repeated Measures ANOVA for G93A vs WT (non-treated)**

Consistent with our previous reports, comparison of the WT vs G93A reveals a significant effect of time (week 8, week 10, week 12, week 14, week 16), group (WT vs G93A), and interaction factors probing that the evolution of the EMG activity in the G93A group is significantly different from that of WT animals.

| **Within Subjects Effects** | | | | | | | | | | | | | |
| --- | --- | --- | --- | --- | --- | --- | --- | --- | --- | --- | --- | --- | --- |
| **Cases** | | **Sphericity Correction** | | **Sum of Squares** | | **df** | | **Mean Square** | | ***F*** | | ***P*** | |
| Weeks |  | None |  | 11000.268 |  | 4.000 |  | 2750.067 |  | 4.850 |  | 0.004 |  |
| Weeks ✻ Group |  | None |  | 7395.621 |  | 4.000 |  | 1848.905 |  | 3.261 |  | 0.024 |  |
| Residuals |  | None |  | 18144.640 |  | 32.000 |  | 567.020 |  |  |  |  |  |
|  | | | | | | | | | | | | | |
| *Note.*  Type II Sum of Squares | | | | | | | | | | | | | |

| **Between Subjects Effects** | | | | | | | | | | | |
| --- | --- | --- | --- | --- | --- | --- | --- | --- | --- | --- | --- |
| **Cases** | | **Sum of Squares** | | **df** | | **Mean Square** | | ***F*** | | ***P*** | |
| Group |  | 11163.549 |  | 1 |  | 11163.549 |  | 9.008 |  | 0.017 |  |
| Residuals |  | 9914.440 |  | 8 |  | 1239.305 |  |  |  |  |  |
|  | | | | | | | | | | | |
| *Note.*  Type II Sum of Squares | | | | | | | | | | | |

Posthoc analyses (Mann-Whitney U test) reveals that the number of SAPs found in TA muscles of G93A is significantly greater when compared to WT mice, starting at 10 weeks-old:

| **Mann-Whitney U test** | | | | | | | |
| --- | --- | --- | --- | --- | --- | --- | --- |
|  | | **U** | |  | | ***P*** | |
| week 8 |  | 17.500 |  |  |  | 0.142 |  |
| week 10 |  | 21.000 |  |  |  | 0.033 |  |
| week 12 |  | 22.000 |  |  |  | 0.021 |  |
| week 14 |  | 24.000 |  |  |  | 0.007 |  |
| week 16 |  | 24.000 |  |  |  | 0.007 |  |
|  | | | | | | | |
| *Note.*  For all tests, the alternative hypothesis specifies that group *TG_Veh* is greater than group *WT_Veh* . | | | | | | | |

**Repeated Measures ANOVA for G93A, treated vs non-treated**

GCN2ib treatment of G93A mice delays the appearance of SAPs, only becoming significant at 14 weeks:

| **Within Subjects Effects** | | | | | | | | | | | | | |
| --- | --- | --- | --- | --- | --- | --- | --- | --- | --- | --- | --- | --- | --- |
| **Cases** | | **Sphericity Correction** | | **Sum of Squares** | | **df** | | **Mean Square** | | ***F*** | | ***P*** | |
| Weeks |  | None |  | 20552.562 |  | 4.000 |  | 5138.140 |  | 9.397 |  | < .001 |  |
| Weeks ✻ Group |  | None |  | 5704.887 |  | 4.000 |  | 1426.222 |  | 2.608 |  | 0.050 |  |
| Residuals |  | None |  | 21872.039 |  | 40.000 |  | 546.801 |  |  |  |  |  |
|  | | | | | | | | | | | | | |
| *Note.*  Type II Sum of Squares | | | | | | | | | | | | | |

| **Between Subjects Effects** | | | | | | | | | | | |
| --- | --- | --- | --- | --- | --- | --- | --- | --- | --- | --- | --- |
| **Cases** | | **Sum of Squares** | | **df** | | **Mean Square** | | ***F*** | | ***P*** | |
| Group |  | 3150.746 |  | 1 |  | 3150.746 |  | 2.951 |  | 0.117 |  |
| Residuals |  | 10676.175 |  | 10 |  | 1067.618 |  |  |  |  |  |
|  | | | | | | | | | | | |
| *Note.*  Type II Sum of Squares | | | | | | | | | | | |

| **Mann-Whitney U test** | | | | | | | | | | | | | | | | | | | | |
| --- | --- | --- | --- | --- | --- | --- | --- | --- | --- | --- | --- | --- | --- | --- | --- | --- | --- | --- | --- | --- |
|  | | | | | | | | | | **U** | | | | |  | | | ***P*** | | |
| week 8 | | | | | | |  | | | 13.500 | | | |  |  |  | | 0.261 | |  |
| week 10 | | | | | | |  | | | 17.500 | | | |  |  |  | | 0.500 | |  |
| week 12 | | | | | | |  | | | 6.000 | | | |  |  |  | | 0.032 | |  |
| week 14 | | | | | | |  | | | 11.000 | | | |  |  |  | | 0.155 | |  |
| week 16 | | | | | | |  | | | 19.000 | | | |  |  |  | | 0.591 | |  |
|  | | | | | | | | | | | | | | | | | | | | |
| *Note.*  For all tests, the alternative hypothesis specifies that group *TG_GCN2iB* is less than group *TG_Veh* . | | | | | | | | | | | | | | | | | | | | |
| **Group Descriptives** | | | | | | | | | | | | | | | | | | |  |  |
|  | | **Group** | | **N** | | **Mean** | | | **SD** | | | **SE** | | **Coefficient of variation** | | | | |  |  |
| week 8 |  | TG_GCN2iB |  | 6 |  | 0.075 | |  | 0.082 | |  | 0.034 |  | 1.099 | | |  | |  |  |
|  |  | TG_Veh |  | 6 |  | 0.390 | |  | 0.636 | |  | 0.260 |  | 1.629 | | |  | |  |  |
| week 10 |  | TG_GCN2iB |  | 6 |  | 4.946 | |  | 3.845 | |  | 1.570 |  | 0.778 | | |  | |  |  |
|  |  | TG_Veh |  | 6 |  | 7.265 | |  | 8.765 | |  | 3.578 |  | 1.206 | | |  | |  |  |
| week 12 |  | TG_GCN2iB |  | 6 |  | 5.698 | |  | 4.109 | |  | 1.677 |  | 0.721 | | |  | |  |  |
|  |  | TG_Veh |  | 6 |  | 42.076 | |  | 23.830 | |  | 9.728 |  | 0.566 | | |  | |  |  |
| week 14 |  | TG_GCN2iB |  | 6 |  | 28.947 | |  | 16.925 | |  | 6.910 |  | 0.585 | | |  | |  |  |
|  |  | TG_Veh |  | 6 |  | 68.733 | |  | 67.517 | |  | 27.564 |  | 0.982 | | |  | |  |  |
| week 16 |  | TG_GCN2iB |  | 6 |  | 41.716 | |  | 24.099 | |  | 9.838 |  | 0.578 | | |  | |  |  |
|  |  | TG_Veh |  | 6 |  | 35.382 | |  | 20.177 | |  | 8.237 |  | 0.570 | | |  | |  |  |
|  | | | | | | | | | | | | | | | | | | |  |  |

**Repeated Measures ANOVA for WT, treated vs non-treated**

No effects were found in GCN2ib-treated WT mice

| **Within Subjects Effects** | | | | | | | | | | | | | |
| --- | --- | --- | --- | --- | --- | --- | --- | --- | --- | --- | --- | --- | --- |
| **Cases** | | **Sphericity Correction** | | **Sum of Squares** | | **df** | | **Mean Square** | | ***F*** | | ***P*** | |
| Weeks |  | None |  | 2.940 |  | 4.000 |  | 0.735 |  | 2.489 |  | 0.063 |  |
| Weeks ✻ Group |  | None |  | 0.254 |  | 4.000 |  | 0.064 |  | 0.215 |  | 0.928 |  |
| Residuals |  | None |  | 9.450 |  | 32.000 |  | 0.295 |  |  |  |  |  |
|  | | | | | | | | | | | | | |
| *Note.*  Type II Sum of Squares | | | | | | | | | | | | | |
| Mauchly's test of sphericity indicates that the assumption of sphericity is violated (*P* < 0.05). | | | | | | | | | | | | | |

| **Between Subjects Effects** | | | | | | | | | | | | | |  |  |
| --- | --- | --- | --- | --- | --- | --- | --- | --- | --- | --- | --- | --- | --- | --- | --- |
| **Cases** | | **Sum of Squares** | | **df** | | **Mean Square** | | | ***F*** | | | ***P*** | |  |  |
| Group |  | 7.661×10^-5^ |  | 1 |  | | 7.661×10^-5^ |  | | 2.970×10^-4^ |  | | 0.987 | |  |
| Residuals |  | 2.063 |  | 8 |  | | 0.258 |  | |  |  | |  | |  |
|  | | | | | | | | | | | | | |  |  |
| *Note.*  Type II Sum of Squares | | | | | | | | | | | | | |  |  |

**EMG evolution in SOL muscle**

**Repeated Measures ANOVA for 4 groups, 5 time points**

2-way mixed ANOVA comparing the evolution of EMG activity at SOL muscle among the two genotypes and treatments (4 groups) detects only a significant effect of the group factor (although the interaction effect is 0.06).

| **Within Subjects Effects** | | | | | | | | | | | | | |
| --- | --- | --- | --- | --- | --- | --- | --- | --- | --- | --- | --- | --- | --- |
| **Cases** | | **Sphericity Correction** | | **Sum of Squares** | | **df** | | **Mean Square** | | ***F*** | | ***P*** | |
| Weeks |  | None |  | 30.116 |  | 4.000 |  | 7.529 |  | 1.568 |  | 0.192 |  |
| Weeks ✻ Group |  | None |  | 105.159 |  | 12.000 |  | 8.763 |  | 1.825 |  | 0.060 |  |
| Residuals |  | None |  | 345.781 |  | 72.000 |  | 4.803 |  |  |  |  |  |
|  | | | | | | | | | | | | | |
| *Note.*  Type II Sum of Squares | | | | | | | | | | | | | |

| **Between Subjects Effects** | | | | | | | | | | | |
| --- | --- | --- | --- | --- | --- | --- | --- | --- | --- | --- | --- |
| **Cases** | | **Sum of Squares** | | **df** | | **Mean Square** | | ***F*** | | ***P*** | |
| Group |  | 66.634 |  | 3 |  | 22.211 |  | 5.005 |  | 0.011 |  |
| Residuals |  | 79.881 |  | 18 |  | 4.438 |  |  |  |  |  |
|  | | | | | | | | | | | |
| *Note.*  Type II Sum of Squares | | | | | | | | | | | |

| **Descriptives** | | | | | | | | | | | | | |
| --- | --- | --- | --- | --- | --- | --- | --- | --- | --- | --- | --- | --- | --- |
| **Weeks** | | **Group** | | **N** | | **Mean** | | **SD** | | **SE** | | **Coefficient of variation** | |
| week 8 |  | TG_GCN2iB |  | 6 |  | 0.068 |  | 0.074 |  | 0.030 |  | 1.084 |  |
|  |  | TG_Veh |  | 6 |  | 0.853 |  | 1.929 |  | 0.787 |  | 2.261 |  |
|  |  | WT_GCN2iB |  | 6 |  | 0.357 |  | 0.806 |  | 0.329 |  | 2.257 |  |
|  |  | WT_Veh |  | 4 |  | 0.018 |  | 0.027 |  | 0.013 |  | 1.460 |  |
| week 10 |  | TG_GCN2iB |  | 6 |  | 0.165 |  | 0.199 |  | 0.081 |  | 1.206 |  |
|  |  | TG_Veh |  | 6 |  | 0.516 |  | 0.713 |  | 0.291 |  | 1.381 |  |
|  |  | WT_GCN2iB |  | 6 |  | 0.496 |  | 0.662 |  | 0.270 |  | 1.335 |  |
|  |  | WT_Veh |  | 4 |  | 0.121 |  | 0.093 |  | 0.047 |  | 0.770 |  |
| week 12 |  | TG_GCN2iB |  | 6 |  | 0.312 |  | 0.676 |  | 0.276 |  | 2.164 |  |
|  |  | TG_Veh |  | 6 |  | 5.011 |  | 6.379 |  | 2.604 |  | 1.273 |  |
|  |  | WT_GCN2iB |  | 6 |  | 0.093 |  | 0.228 |  | 0.093 |  | 2.449 |  |
|  |  | WT_Veh |  | 4 |  | 0.027 |  | 0.021 |  | 0.010 |  | 0.790 |  |
| week 14 |  | TG_GCN2iB |  | 6 |  | 1.265 |  | 1.433 |  | 0.585 |  | 1.133 |  |
|  |  | TG_Veh |  | 6 |  | 2.991 |  | 4.573 |  | 1.867 |  | 1.529 |  |
|  |  | WT_GCN2iB |  | 6 |  | 0.129 |  | 0.118 |  | 0.048 |  | 0.922 |  |
|  |  | WT_Veh |  | 4 |  | 1.286 |  | 1.994 |  | 0.997 |  | 1.551 |  |
| week 16 |  | TG_GCN2iB |  | 6 |  | 3.365 |  | 3.078 |  | 1.257 |  | 0.915 |  |
|  |  | TG_Veh |  | 6 |  | 1.346 |  | 1.911 |  | 0.780 |  | 1.420 |  |
|  |  | WT_GCN2iB |  | 6 |  | 0.124 |  | 0.263 |  | 0.107 |  | 2.115 |  |
|  |  | WT_Veh |  | 4 |  | 0.000 |  | 0.000 |  | 0.000 |  | NaN |  |
|  | | | | | | | | | | | | | |

Bonferroni post hoc test on the group factor reveals that overall (time factor) there is no difference between treating or non-treating WT mice, non-treated G93A mice show significantly more EMG activity than non-treated or treated WT mice and although difference does not reach significance threshold, treated G93A mice show less activity than non-treated G93A mice.

| **Post Hoc Comparisons - Group** | | | | | | | | | | | |
| --- | --- | --- | --- | --- | --- | --- | --- | --- | --- | --- | --- |
|  | |  | | **Mean Difference** | | **SE** | | **t** | | ***P*_bonf_** | |
| TG_GCN2iB |  | TG_Veh |  | -1.108 |  | 0.544 |  | -2.038 |  | 0.339 |  |
|  |  | WT_GCN2iB |  | 0.795 |  | 0.544 |  | 1.462 |  | 0.966 |  |
|  |  | WT_Veh |  | 0.745 |  | 0.608 |  | 1.224 |  | 1.000 |  |
| TG_Veh |  | WT_GCN2iB |  | 1.903 |  | 0.544 |  | 3.500 |  | 0.015 | * |
|  |  | WT_Veh |  | 1.853 |  | 0.608 |  | 3.047 |  | 0.042 | * |
| WT_GCN2iB |  | WT_Veh |  | -0.051 |  | 0.608 |  | -0.083 |  | 1.000 |  |
|  | | | | | | | | | | | |
| * *P* < 0.05 | | | | | | | | | | | |
| *Note.*  P-value adjusted for comparing a family of 6 | | | | | | | | | | | |
| *Note.*  Results are averaged over the levels of: Weeks | | | | | | | | | | | |

**Supplementary Statistical Analysis in Figure 1k-m**

**k.** Time (*F*_10, 180_ = 35.92, *P<*0.001), Condition (*F*_3, 18_ = 34.51, *P<*0.001), Time x Condition Interaction (*F*_30, 180_= 14.46, *P<*0.001). **l.** Time (*F*_4, 52_ = 7.377, *P<*0.001), Time x Condition interaction (*F*_8, 52_ = 2.521, *P<* 0.05). Differences apply to 16 weeks in comparison to WT+veh. **m.** Time (*F*_10, 180_ = 79.71, *P<*0.001), Time x Condition interaction (*F*_30, 180_ = 4.264, *P<*0.001).
