# Supplementary material for: GCN2 inhibition reduces mutant SOD1 clustering and toxicity and delays disease progression in an amyotrophic lateral sclerosis mouse model
Source: Transl Neurodegener. 2024 Sep 20;13:49. doi: 10.1186/s40035-024-00441-w (PMC11414287; doi:10.1186/s40035-024-00441-w)
Supplement: Supplementary file 2 — Additional file 2. Materials and Methods. [file 40035_2024_441_MOESM2_ESM.docx]

## Additional file 2

## Materials and Methods

### Animals

Wild-type (WT) (C57BL/6J, strain #000664) and transgenic (G93A) (B6.Cg-Tg (SOD1*G93A) 1Gur/J, strain #004435) mice (carrying the human mutant SOD1G93A transgene [1])were acquired from Jackson Laboratory (ME, USA). G93A mice were crossed with WT females at pathogen-free CIMA facilities as previously described [2]. Cages were at 21 °C in a humidity-controlled environment on a 12-hour light/dark cycle with wood shavings and sterile paper tissue. Transgenic mice were identified by PCR analysis using primers and following cycling protocols recommended by Jackson Laboratory (https://www.jax.org/strain/004435). GCN2ib (dissolved in an aqueous solution containing 0.5% methylcellulose and 2% DMSO) was administrated intraperitoneally at a dose of 10 mg.kg^-1^ twice daily from week 6.

### Mouse behavioral tests

Mouse motor phenotype was evaluated with the hanging wire test as described elsewhere [2]. Additionally, an 8-point scale clinical score; from 8 (normal functions, no sign of disease onset) to 0 (unable to right itself within 30 seconds) [3] was used to monitor disease progression.

### Electromyography recording and analysis

Longitudinal spontaneous electromyographic (EMG) activity was assessed by recording muscle activity in five different sessions at 8, 10, 12, 14, and 16 weeks. The EMG signal was amplified 10000 times and passed through a bandpass filter with low and high cut-off frequency settings of 10 and 1000 Hz, respectively (DAM70 differential amplifier, WPI). Amplified signal was digitized with a Power 1401, 625 kHz, 16-bit ADC (Cambridge Electronic Design) converter under the control of Spike2 software that also served to store and review the data. After that, 15 s length segments of recording per side and muscle were selected and analyzed using custom-made scripts developed under Matlab. EMG activations from recordings were detected by thresholding the signal at 4.5 times the 95 quantiles of the EMG activity recorded from WT animals (to assess the robustness of this approach, several thresholds were investigated showing no effect; data not shown). The number of activations per second was used to assess the amount of spontaneous EMG activity in the animals as a marker of the neurodegenerative process.

### Tissue processing and Immunohistochemistry

Mice were transcardially perfused and the spinal cord was extracted and processed following Marlin et al. protocols [2]. For immunohistochemistry experiments spinal cord sections were first tempered for 1-2 hours at room temperature (RT). After three washes with PBS at pH 7.4 (5 minutes each), antigen retrieval was performed. First, sections were covered with 0.1 M citrate buffer pH 6.0 (citric acid (Ref: 25.127-5, Sigma-Aldrich) and sodium citrate tribasic dihydrate (C6H5Na3O7.2H2O) (Ref: S-4641, Sigma) for 10 minutes at RT. Then, sections were placed into previously heated (at 90 °C) Coplin jars with 0.1M citrate buffer pH 6.0, that were introduced into a 97 °C bath for 30 minutes. After this step, the Coplin jar was tempered (30 minutes) at RT. After three washes with PBS, endogenous peroxidases were inactivated by incubating sections with methanol (PanReac, AppliChem, Barcelona, Spain) and hydrogen peroxide (H_2_O_2_) (Sigma-Aldrich) solution (91.18% methanol and 2.74% H_2_O_2_ in sterile water, stored at 4 °C) for 30 minutes. Three additional PBS washes were performed next. A box enclosing all spinal cord sections in the slides was done with a wax pencil (Liquid Blocker Super Pap Pen) (Electron Microscopy Sciences, USA). Sections in the box were first incubated with blocking solution (4% Normal Goat Serum (NGS) (Jackson ImmunoResearch), 0.05% Triton X-100, and 4% Bovine Serum Albumin (BSA) (Sigma-Aldrich) in PBS) for 40 minutes and next, with primary antibodies (Table 1) diluted in blocking solution (in a humid chamber during 72 hours at 4 °C). After this incubation, samples were tempered for 1h at RT and washed thrice with PBS. Then, samples were incubated with secondary biotinylated antibodies (Table 1) in blocking solution for 90 minutes at RT. After three washes with PBS, samples were incubated with Streptavidin (HRP, Ab7403, Abcam) (1:5000 in PBS) for 90 minutes. Samples were then washed thrice with PBS and incubated with 0.05% diaminobenzidine (Sigma-Aldrich), 0.03% H_2_O_2_ and Tris buffer 0.05 M in PBS pH 7.6. After three final PBS washes, sections were dried overnight and dehydrated in toluene (Scharlab, Spain) for 12 minutes. Finally, sections were covered with DPX mounting medium fast (PanReac, AppliChem).

### Plasmids

pCAGGS-SOD1 (WT and mutants G85R, G93A) mCherry (Ch)-tagged and untagged versions were already described [4].

Generation of CRISPR-SpCas9 plasmids carrying guides to target human and rat ISR kinases (PKR, PERK, HRI, and GCN2) (Tables 2 and 3, Figure S3): Two different single guide RNAs (sgRNAs) primers targeting each one of the ISR kinase genes (human and rat) were designed using Benchling. The corresponding gene sequence was downloaded from GeneBank, and the Cas9 Protospacer Adjacent Motif (PAM) sequence NGG (any nucleotide, guanine, guanine) required for *S. pyogenes* was considered (Table 2). sgRNAs primers were inserted into the plasmid pX459 (Addgene, Watertown, MA).

All plasmids were confirmed by Sanger sequencing.

### Cell lines and primary neuronal cultures

Human embryonic kidney (HEK293) and McA-RH7777 (RH7777) cells (from ATCC), were grown and maintained in Dulbecco’s Modified Eagle’s Medium (DMEM) (Sigma-Aldrich) supplemented with 10% Fetal Bovine Serum (FBS), 1% Penicillin-Streptomycin-Glutamine (Gibco), in 5% CO_2_ and 37 °C inside a humidified incubator [2]. Rat cortical primary neurons were established from Sprague Dawley rat embryos (embryonic day 20) according to protocols already described [5].

### Calcium phosphate transfection of cell line and primary neurons

HEK293 cells were transfected with DNA-calcium phosphate mix as described [5]. Briefly, cells were plated in 24-well plates (with coverslips for immunofluorescence) or 6-well plates (for western blot, WB) to reach 50% confluence. One hour before transfection, growth medium was replaced by DMEM (Lonza). Meanwhile, the DNA-calcium phosphate transfection buffer was prepared by mixing the DNA-containing solution (2 µg of plasmid DNA/well of 24-well plates or 8 µg of plasmid DNA/well of 6-well plates, 125 mM CaCl2 (Sigma) and the HEPES buffer solution, pH 7.02 (137 mM NaCl (Sigma-Aldrich), 5 mM KCl, 0.7 mM Na2HPO4*7H2O, 7.5 mM D-(+)-Glucose (dextrose) (Sigma) and 21 mM HEPES (Sigma)). The mix was incubated at room temperature (RT) for 20 minutes and added to each well [5]. Cells were incubated for 4 hours until a thin DNA-calcium phosphate precipitate was visible under the microscope. Finally, the transfection mix was replaced with growth medium and cells were placed back into the incubator.

Transfection of RH7777 cells was performed with Lipofectamine 2000 (Invitrogen) following recommended protocols.

Primary cortical neurons in 24-well plates with/without coverslips (for immunofluorescence experiments or longitudinal survival analysis, respectively) were transfected at 5 days *in vitro* with a calcium phosphate protocol [6]; 1 h prior transfection neuronal growth medium was replaced by DMEM (Lonza) with 0.001% gentamicin/1 mM Kynurenic acid (KY), pH 7.4. The DNA‑calcium phosphate mix (per well: 2μg of plasmid DNA, 125 mM CaCl2 (Sigma) and HEPES buffer solution at pH range (6.8–7.2) (137 mM NaCl (Sigma-Aldrich), 5 mM KCl, 0.7 mM Na2HPO4*7H2O, 7.5 mM D-(+)-Glucose (dextrose) (Sigma) and 21 mM HEPES (Sigma)) was prepared, incubated for 20 minutes at RT and added to each well. Neurons were incubated until a thin DNA‑calcium phosphate precipitate was observed (45-60 minutes). Then, the medium was replaced with shock solution (50% (V/V) HEPES buffer solution pH 7.4, 10% (V/V) DMEM/KY pH 7.4, and 2% (V/V) DMSO (Sigma)) and rapidly washed twice with DMEM/KY and once with DMEM supplemented with 0.001% gentamicin. Neurons were finally left with neuronal growth media (Neurobasal medium supplemented with 0.5% FBS, 1% GlutaMAX (Gibco), 2% B27 (Gibco), 0.1 mg/ml gentamycin (Life Technologies, Carlsbad, CA) and 2.5 mg/ml fungizone (Gibco)) into the incubator.

For SOD1 (WT, G93A, and G85R) intracellular distribution and foci formation studies, HEK293 cells were transiently transfected with pCAGGs-SOD1 (WT, G93A, or G85R) plasmids. To address the effect of ISR modulation HEK293 cells were either co-transfected with CRISPR-Cas9 plasmids to target the human ISR kinases or treated with ISR modulators (see below) added 10 minutes after transfection. Ninety-six hours post-transfection treated and untreated cells were fixed with 4% paraformaldehyde (Fluka, Thermo Fisher Scientific, Waltham, MA) and 4% sucrose in PBS (PFA) for immunofluorescence (IF) staining and microscopy analysis.

For analyzing the effect of ISR modulation in the intraneuronal distribution of SOD1, neurons were transfected with untagged pCAGGs-SOD1 (WT, G93A) plasmids and treated with ISR modulators. For addressing the role of ISR modulation in neuronal survival by longitudinal survival analysis, neurons were transfected with pCAGGS-SOD1-G93A mCherry-tagged (G93ACh) or pCAGGs-mCherry (Ch) in the case of treatments with ISR modulators or co-transfected with CRISPR-Cas9 plasmids to target rat GCN2 kinase.

### Pharmacological induction and modulation of the ISR

PERK pharmacological inducer Thapsigargin (Thap)(Applichem) and the ISR modulators ISRIB (Sigma), GSK2606414 (PERKib)[7] (Toronto Research Chemicals, North York, Canada), A92 [8](Axon MedChem) and GCN2ib [9](WUXi App Tec, Hong Kong), were dissolved in dimethyl sulfoxide (DMSO) (Sigma) and stored at −80  °C. GCN2 activator (Histidinol-Dihydrochloride (HisOH) (Sigma-Aldrich)), HRI activator (Sodium Arsenite_ARS) (Sigma-Aldrich), Oligomycin, and PKR activator poly (I:C) (Sigma-Aldrich) were dissolved in sterile water and stored at -20 °C until used.

Naïve HEK293 cells were treated with Thapsigargin 200 nM (2 hours), HisOH 2 mM (4 hours), ARS 200 µM (1 hours) or 10 µg/well poly(I:C) (24 hours), either in presence or absence of ISR modulators ISRIB (500 nM), GSK (500 nM), GCN2ib (1 µM) or DMSO as control. Both inducers and modulator treatments were added at the same time. Protein samples were collected in 200 µL Laemmli buffer, boiled at 95 °C for 10 minutes, and stored at -20 °C until WB analysis.

To test the effect of ISR modulators in SOD1 aggregation, they were added 10 minutes after transfection both in the case of transfected HEK293 cells and primary neurons. Cells/neurons were fixed with PFA (96 hours post-transfection) for IF staining and microscopy analysis.

To address the effect of GCN2 inhibition in neuronal survival, transfected neurons were treated with A92 (0.5 μm, 1 μm) or vehicle 16 hours post-transfection, added before the first image acquisition (T1) in the longitudinal survival analysis.

### Western blot (WB) (SDS-PAGE)

Protein samples for WB analysis were prepared from monolayer cell cultures. Cells were washed twice with ice-cold phosphate-buffered saline (PBS) and collected in 200 μl of Laemli buffer [50 mM Tris pH 6.8 (Bio-Rad, Hercules, CA), 100 mM dithiothreitol (Sigma), 2% sodium dodecyl sulfate (SDS) (Bio-Rad), 10% glycerol (Sigma), 0.01% bromophenol blue (Merck, Darmstadt, Germany), Phos-STOP phosphatase inhibitor cocktail (Roche, Basel, Switzerland), 17.5 mM β-glycerol phosphate (Sigma), and Complete protease inhibitor cocktail (Roche)]. Total protein extracts were transferred to clean 1.5 ml polypropylene tubes and boiled at 95 °C for 10 minutes.

### Immunofluorescence (IF)

Cells and neurons growing on coverslips were fixed 96 hours after transfection with PFA for 9 minutes. Coverslips were then washed twice with PBS and kept at 4 °C. For IF experiments, cells were first permeabilized with 0.1%Triton x-100 in PBS (PBT) for 20 minutes, followed by a 20-minute incubation with 1 M glycine (BioRad) to quench autofluorescence and 1-hour incubation with blocking solution (3% BSA (Merck-Millipore) in PBT). Next, cells were incubated with primary and secondary antibodies in blocking solution for 2 hours each, at RT. After staining with DAPI (Sigma), coverslips were mounted over glass slides in mounting medium (25 mg DABCO Sigma per milliliter (ml) of Permafluor (Thermo Scientific), dried for 30 minutes at 37 °C and kept at 4 °C until microscope visualization (see further details of this protocol in [5]). Images were acquired with 40x and 63x objectives on a Zeiss Axiovert 200M Fluorescence microscope (Zeiss, Oberkochen, Germany). Both acquisition and processing were conducted with MetaMorph Microscopy Automation and Image Analysis Software (Molecular Devices, San Jose, CA). A minimum of 10 images per condition were acquired to reach a minimum of n = 100 cells per condition in each independent experiment.

### Microscopy for image acquisition

Images from neurons and HEK293 cells were acquired with 40X and 63X objectives on a Zeiss Axiovert 200M fluorescence microscope with MetaMorph Microscopy Automation and Image Analysis Software (Molecular Devices). A Zeiss LSM-800 confocal microscope with Zen System 2.3 software (Zeiss) and 63X objective was used for the acquisition of confocal images. Each confocal file was acquired as a series of 20-40 Z-stack images spaced approximately 15-25 μm.

#### Automated image acquisition

Primary transfected neurons in 24-well plates were subjected to longitudinal survival analysis by automated microscopy [5][6][4] in a Zeiss Observer Z1 microscope, with a chamber to maintain stable temperature and CO_2_ conditions (37 °C, 5% CO_2_). Typically, 3-4 wells per condition and 2 plates (replicates) per experiment were imaged. Specifically, 10 non-overlapping positions on each well were randomly selected on the first day and imaged with 10X long-distance objectives. The same positions were imaged every 24 hours for 7-8 consecutive days with the Zen System software (Zeiss) [5].

#### Colorimetric immunohistochemistry images

Images from colorimetric immunohistochemistry in spinal cord samples were acquired with an Aperio CS2 microscope (20X magnification, Aperio Scan Scope software) (Leica).

### Image Processing and Statistics

#### Image processing

Quantification of individual cells per field and fluorescence intensity in single HEK293 cells/neurons were analyzed with Metamorph Meta Imaging Series 7.7 software in images from an Axiovert 200M microscope (40X magnification). The final average fluorescence intensity values were the result of subtracting the background signal from the fluorescence intensity measured in an area corresponding to the whole cell cytoplasm or neuronal soma. Those images were also exported as TIFF for unbiased analysis of SOD1 intracellular pattern distribution with FociCount (see below).

#### Semiautomated Image processing for clustering analysis (FociCount)

To avoid quantification bias, we developed a customized FIJI-based semiautomated quantification tool, FociCount. FociCount 1) automatically enhances the image contrast of epifluorescence images, allowing the manual determination of the perimeter of individual cells; then, 2) it detects and counts individual foci using the function *find maxima* and, finally 3) uses the algorithm *growing* region to determine area and average fluorescence intensity for each focus. Quantitation of FociCount-derived data allows us to score two main parameters: a) the number of foci per cell and b) the fraction of fluorescence signal in foci.

#### Motoneurons counting

ChAT-positive motoneurons were counted in 5 lumbar spinal cord sections (L1 to L5) (left and right ventral horns). Images were selected with Aperio Image Scope (Leica) taking as reference the Mouse Spinal Cord atlas (Allen Brain Atlas, Allen Institute for Brain Science). Analysis was performed with the open-source Fiji (ImageJ) software.

#### Statistics

To estimate survival times for individual neurons, image analysis was performed with a Matlab-based semi-automated ad hoc program [5]; the program opens the same neuronal field sequentially at all the experimental times. At the initial time, neurons are selected and numbered. For the rest of the time series, the user determines the presence of each neuron by comparing the images with the previous time. Dead neurons are identified and categorized as uncensored events. Neurons that survive until the end of the experiment are categorized as censored events. The data is exported to Excel and survival analyses are performed with STATA 12. Nelson-Aalen cumulative hazard functions were used to plot differences in the cumulative risk of death among experimental groups. As long as the proportional hazard assumption is fulfilled (Shoenfeld residual-based test evaluation or graphical assessment), differences are analyzed with a log-rank test and clustered Cox regression models. A clustered Cox regression analysis of neurons co-existing in the same well is performed to improve the accuracy of the test and, since all experiments were repeated at least thrice with one or two plates 24-well plates per experiment, the variability in the baseline toxicity between experiments was adjusted by stratifying the Cox model for each plate.

Experiments with mice were conducted in a randomized and blinded manner, although the motor phenotype progression in G93A transgenic mice was noticeable over time. Shapiro-Wilk normality test was used to address the normal distribution. In the case of non-normality, Kruskal-Wallis test was used for multiple comparisons. Only in the case of *P<*0.05, Sidak and Holm-Sidak post-hoc test were used. Additionally, one-way ANOVA, two-way ANOVA (two-way repeated measures (mixed) ANOVA in the case of matched values) and three-way ANOVA with post-hoc tests (Tukey, Mann-Whitney U and Bonferroni) were used. Only p values less than 0.05 were considered statistically significant. Post hoc tests were run only if F achieved the necessary level of statistical significance (*P<* 0.05). Statistical analyses for longitudinal EMG activity have been included as Supplementary Material. Data analyses were carried out with Microsoft Excel 2010 (Microsoft Corp.), GraphPad Prism 8 software (GraphPad Software Inc.), StataIC 12, and Matlab.

### Tables

| **Table 1. List of antibodies** |  |  |
| --- | --- | --- |
| Antibody | Provider (reference) | Application |
| Anti-Human SOD1 | OriGene  TA500495 | WB (1:1,000)  IF (1:1,000) |
| Anti-Human SOD1  Purified (C4F6) | MEDIMABS  MM-0070-2-P | IF (1:100) |
| G3BP2 | Cell Signaling  31799 | IF (1:1,000) |
| Total eIF2a | Santa Cruz  sc-11386 | WB (1:1,000) |
| Phospho eIF2a (Ser51) | Cell Signaling  9721 | WB (1:1,000) |
| Total PKR | Cell Signaling  3072S | WB (1:1,000) |
| Phospho PKR  (T446) | Abcam  Ab32036 | WB (1:1,000) |
| PERK | Cell Signaling  3192 | WB (1:1,000) |
| Total GCN2 | Cell Signaling  3302 | WB (1:1,000) |
| Phospho GCN2  (T899) | Abcam  ab7583 | WB (1:1,000) |
| ATF4 (D4B8) | Cell Signaling  11815 | WB (1:1,000) |
| MAP2 | Abcam  Ab5392 | IF (1:500) |
| GAPDH (14C10) | Cell Signaling  2118 | WB (1:1,000) |
| α-Tubulin | Sigma Aldrich  T6074 | WB (1:10,000) |
| Anti-choline acetyltransferase (ChAT) | Merck Millipore  AB144P | IF (1:500)  IHC |
| Biotin-SP  (long spacer) AffiniPure  F (ab')₂Fragment DonkeyAnti-Goat IgG (H+L) Donkey | Jackson ImmunoResearch  705-066-147 | IF (1:500) |
| AntiMouse IgG-HRP | Sigma  A0168 | WB (1:10,000) |
| AntiRabbit IgG-HRP | Sigma  A0545 | WB (1:10,000) |
| Alexa 405 anti-chicken IgG | Abcam  Ab175674 | IF (1:500) |
| Alexa 488 anti-mouse IgG | Invitrogen  A-11001 | IF (1:500) |
| Cy3-conjugated anti-rabbit IgG | Jackson Immunoresearch  (115-165-003) | IF (1:500) |
| Cy5-conjugated anti-rabbit IgG | Jackson Immunoresearch  (115-175-144) | IF (1:500) |
| Biotin-conjugated anti-mouse IgG | Jackson Immunoresearch  (115-065-003) | IF (1:500) |
|  |  |  |

WB: western blot. IF: immunofluorescence. IHC: Immunohistochemistry.

| Table 2. Cloning Primers for Human ISR Kinases CRISPR/Cas9 Guides | | | | | |
| --- | --- | --- | --- | --- | --- |
| ID | SENSE | CLONING PRIMER FW | CLONING PRIMER RV | AA SEQ | AA SITE |
| GUIDE 1 HRI EX1 | + | CACCCGGGGTCCGCAAGCGCGAAG | AAACCTTCGCGCTTGCGGACCCCG | SGVRKRE | 6-12 |
| GUIDE 2 HRI EX7 | - | CACCATGAACATGTTCTATCCACG | AAACCGTGGATAGAACATGTTCAT | IEHVH | 234-238 |
| GUIDE 1 PKR EX5 | + | CACCATTATGAACAGTGTGCATCG | AAACCGATGCACACTGTTCATAAT | NYEQCAS | 115-123 |
| GUIDE 2 PKR EX7 | - | CACCCGTAGTAGCAAAAGAACCAG | AAACCTGGTTCTTTTGCTACTACG | SGSFATT | 179-185 |
| GUIDE 1 PERK EX1 | + | CACCGAGACAGAGTTGCGACCGCG | AAACCGCGGTCGCAACTCTGTCTC | ETELRP | 95-101 |
| GUIDE 2 PERK EX5 | + | CACCATGGCATTCAGTAAGAAGGG | AAACCCCTTCTTACTGAATGCCAT | MAFSKK | 320-326 |
| GUIDE 1 GCN2 EX 1 | - | CACCCGTTGCGGGTAGCTCTCCGG | AAACCCGGAGAGCTACCCGCAACG | PESYPQ | 16-22 |
| GUIDE 2 GCN2 EX 3 | + | CACCACTGGCCAAGAAACACTGTG | AAACCACAGTGTTTCTTGGCCAGT | LAKKHC | 112-118 |

| Table 3. Cloning Primers for Rat GCN2 CRISPR/Cas9 Guides | | | | | |
| --- | --- | --- | --- | --- | --- |
| ID | SENSE | CLONING PRIMER FW | CLONING PRIMER RV | AA SEQ | AA SITE |
| GUIDE 1 GCN2  EX 1 | - | AAACTGGAGGCGATTTACGGCTCGG | CACCCCGAGCCGTAAATCGCCTCCA | LEAIYGSD | 30-37 |
| GUIDE 2 GCN2 EX 3 | + | CACCGAGAACTGGCCAAAAAGCAGTG | AAACCACTGCTTTTTGGCCAGTTCTC | EELAKKQC | 111-118 |
| GUIDE 3 GCN2 EX 8 | - | AAACCGGGCAGCTTTGTCTTGTTGTC | CACCGACAACAAGACAAAGCTGCCCG | TGSFVLLY | 304-311 |

###

## References

[1] M. E. Gurney *et al.*, “Motor neuron degeneration in mice that express a human Cu,Zn superoxide dismutase mutation,” *Science (1979)*, vol. 264, no. 5166, pp. 1772–1775, 1994, [Online]. Available: http://www.ncbi.nlm.nih.gov/entrez/query.fcgi?cmd=Retrieve&db=PubMed&dopt=Citation&list_uids=8209258

[2] E. Marlin *et al.*, “Pharmacological inhibition of the integrated stress response accelerates disease progression in an amyotrophic lateral sclerosis mouse model.,” *Br J Pharmacol*, Oct. 2023, doi: 10.1111/bph.16260.

[3] C. Zhou, C.-P. Zhao, C. Zhang, G.-Y. Wu, F. Xiong, and C. Zhang, “A method comparison in monitoring disease progression of G93A mouse model of ALS.,” *Amyotroph Lateral Scler*, vol. 8, no. 6, pp. 366–372, Dec. 2007, doi: 10.1080/17482960701538759.

[4] R. Bugallo *et al.*, “Fine tuning of the unfolded protein response by ISRIB improves neuronal survival in  a model of amyotrophic lateral sclerosis.,” *Cell Death Dis*, vol. 11, no. 5, p. 397, May 2020, doi: 10.1038/s41419-020-2601-2.

[5] I. Íñigo-Marco *et al.*, “E46K α-synuclein pathological mutation causes cell-autonomous toxicity without altering protein turnover or aggregation,” *Proc Natl Acad Sci U S A*, vol. 114, no. 39, pp. E8274–E8283, 2017, doi: 10.1073/pnas.1703420114.

[6] R. Vinueza-Gavilanes *et al.*, “N-terminal acetylation mutants affect alpha-synuclein stability, protein levels and neuronal toxicity,” *Neurobiol Dis*, vol. 137, p. 104781, Apr. 2020, doi: 10.1016/j.nbd.2020.104781.

[7] J. M. Axten *et al.*, “Discovery of 7-methyl-5-(1-{[3-(trifluoromethyl)phenyl]acetyl}-2,3-dihydro-1H-indol-5-yl)-7H-p yrrolo[2,3-d]pyrimidin-4-amine (GSK2606414), a potent and selective first-in-class inhibitor of protein kinase R (PKR)-like endoplasmic reticulum kinase (PERK),” *J Med Chem*, vol. 55, no. 16, pp. 7193–7207, 2012, doi: 10.1021/jm300713s.

[8] A. Nakamura *et al.*, “Inhibition of GCN2 sensitizes ASNS-low cancer cells to asparaginase by disrupting the amino acid response,” *Proc Natl Acad Sci U S A*, vol. 115, no. 33, pp. E7776–E7785, 2018, doi: 10.1073/pnas.1805523115.

[9] E. L. Spaulding *et al.*, “The integrated stress response contributes to tRNA synthetase-associated  peripheral neuropathy.,” *Science*, vol. 373, no. 6559, pp. 1156–1161, Sep. 2021, doi: 10.1126/science.abb3414.
